# Secernin‐2 Stabilizes Histone Methyltransferase KMT2C to Suppress Progression and Confer Therapeutic Sensitivity to PARP Inhibition in Triple‐Negative Breast Cancer

**DOI:** 10.1002/advs.202413280

**Published:** 2025-01-21

**Authors:** Min‐Ying Huang, Jia‐Yang Cai, Shao‐Ying Yang, Qian Zhao, Zhi‐Min Shao, Fang‐Lin Zhang, Yin‐Ling Zhang, A‐Yong Cao, Da‐Qiang Li

**Affiliations:** ^1^ Shanghai Cancer Center and Institutes of Biomedical Sciences Shanghai Medical College Fudan University Shanghai 200032 China; ^2^ Department of Oncology Shanghai Medical College Fudan University Shanghai 200032 China; ^3^ Cancer Institute, Shanghai Medical College Fudan University Shanghai 200032 China; ^4^ Department of Breast Surgery Shanghai Medical College Fudan University Shanghai 200032 China; ^5^ Shanghai Key Laboratory of Breast Cancer Shanghai Medical College Fudan University Shanghai 200032 China; ^6^ Shanghai Key Laboratory of Radiation Oncology Shanghai Medical College Fudan University Shanghai 200032 China

**Keywords:** DNA damage response, lysine methyltransferase, PARP inhibition, triple‐negative breast cancer, tumor suppressor

## Abstract

Triple‐negative breast cancer (TNBC) is a difficulty and bottleneck in the clinical treatment of breast cancer due to a lack of effective therapeutic targets. Herein, we first report that secernin 2 (SCRN2), an uncharacterized gene in human cancer, acts as a novel tumor suppressor in TNBC to inhibit cancer progression and enhance therapeutic sensitivity to poly(ADP‐ribose) polymerase (PARP) inhibition both in vitro and in vivo. SCRN2 is downregulated in TNBC through chaperone‐mediated autophagic degradation, and its downregulation is associated with poor patient prognosis. Moreover, SCRN2 impedes the proteasomal degradation of histone‐lysine N‐methyltransferase 2C (KMT2C) by recruiting Bcl2‐associated athanogene 2 to block the interaction of KMT2C with E3 ubiquitin‐protein ligase CHIP. Consistently, SCRN2 transcriptionally activates Bcl2‐modifying factor by amplifying histone H3 monomethylation at lysine 4 at its enhancer, thereby inducing intrinsic apoptosis. Notably, KMT2C knockdown restores the impaired TNBC progression caused by SCRN2 overexpression both in vitro and in vivo. Furthermore, SCRN2 decreases the expression of key DNA repair‐related genes and induces endogenous DNA damage, thus conferring therapeutic sensitivity of TNBC cells to PARP inhibition.   Collectively, these findings identify SCRN2 as a novel suppressor of TNBC, reveal its mechanism of action, and highlight its potential role in TNBC therapy.

## Graphical abstract

Triple‐negative breast cancer (TNBC) is a difficulty and bottleneck in the clinical treatment of breast cancer. This study defines SCRN2 as a novel tumor suppressor in TNBC to suppress cancer progression and enhance PARP inhibitor sensitivity through stabilizing KMT2C and reveals SCRN2 as a potential biomarker and therapeutic target for TNBC.

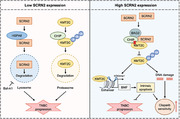

## 1 Introduction

Triple‐negative breast cancer (TNBC) is a specific subtype of breast cancer that lacks the expression of estrogen and progesterone receptors and human epidermal growth factor receptor 2 (HER2), and exhibits the most aggressive clinicopathological features with early distant metastasis, relapse, and the worst outcomes.^[^ ^1^, ^2^ ^]^ Cytotoxic chemotherapy is the mainstay of systemic treatment for this disease owing to its insensitivity to classical endocrine and HER2‐targeted therapies,^[^ ^1^, ^2^ ^]^ however, less than 30% of TNBC patients completely responded to standard chemotherapy.^[^ ^3^ ^]^ Encouragingly, newly emerging targeted agents, such as poly (ADP‐ribose) polymerase (PARP) inhibitors (PARPi), are expanding the therapeutic options for this lethal disease.^[^ ^1^, ^2^ ^]^ However, the development of therapeutic resistance to PARPi has resulted in an unsatisfactory outcome. Therefore, it is of pivotal importance to identify new biomarkers that could be used to select suitable patients who may benefit the most from PARPi treatment and to discover new therapeutic targets for TNBC.

To address these issues, we analyzed our recently published TNBC proteomic dataset^[^ ^4^ ^]^ and found that secernin‐2 (SCRN2) was downregulated in TNBC, which was associated with poor patient prognosis. SCRN2 is a member of the secernin family, which also includes SCRN1 and SCRN3.^[^ ^5^, ^6^ ^]^ Emerging evidence has shown that SCRN1 acts as a regulator of exocytosis,^[^ ^7^ ^]^ axonal regeneration,^[^ ^8^ ^]^ and dynamic endoplasmic reticulum remodeling,^[^ ^9^ ^]^ and that its deregulation contributes to the pathogenesis of Alzheimer's disease and human cancer.^[^ ^8^, ^10^, ^11^, ^12^, ^13^, ^14^, ^15^ ^]^ SCRN3 also involved in thermal nociception in response to inflammatory stimuli.^[^ ^16^ ^]^ Comparatively, SCRN2 is a poorly characterized gene. Available evidence shows that SCRN2 possesses an electrophilic N‐terminal glyoxylyl (Glox) group that acts as an electrophilic cofactor.^[^ ^5^ ^]^ In addition, SCRN2 is a potential candidate susceptibility gene for the Alzheimer's disease and respiratory infectious diseases.^[^ ^17^, ^18^ ^]^ However, the functional and mechanistic roles of SCRN2 in human cancer remain unclear.

Histone lysine N‐methyltransferase 2C (KMT2C; also known as MLL3), a member of the mixed‐lineage leukaemia (MLL) family, possesses lysine methyltransferase activity responsible for the mono‐methylation of histone H3 at lysine 4 (H3K4me1) at gene enhancers.^[^ ^19^, ^20^, ^21^ ^]^ KMT2C functions as a putative tumor suppressor in various human cancers, including breast cancer.^[^ ^19^, ^22^, ^23^ ^]^ Functional assays using various model systems have demonstrated that KMT2C loss accelerates the initiation, growth, and metastasis of breast cancer and affects its responsiveness to antitumor drugs.^[^ ^24^, ^25^, ^26^, ^27^ ^]^ Despite these advances, the mechanisms underlying the regulation of its protein stability in human cancers remain largely unknown.

In this study, we provide the first evidence that SCRN2 functions as a novel tumor suppressor in TNBC to block cancer progression and enhance therapeutic response to PARP inhibition. Mechanistic investigations reveal that SCRN2 is subjected to lysosomal degradation in TNBC through chaperone‐mediated autophagy (CMA) pathway and stabilizes KMT2C by blocking its proteasomal degradation to transcriptionally activate pro‐apoptotic factor BMF. Moreover, SCRN2 is an element of endogenous DNA damage response and confers cellular sensitivity to PARP inhibition. Together, these findings verify the tumor suppressor role of SCRN2 in TNBC and identify SCRN2 as a potential therapeutic target for TNBC.

## 2 Results

### 2.1 SCRN2 is Downregulated in TNBC that is Associated with Poor Patient Prognosis

**Figure 1:**
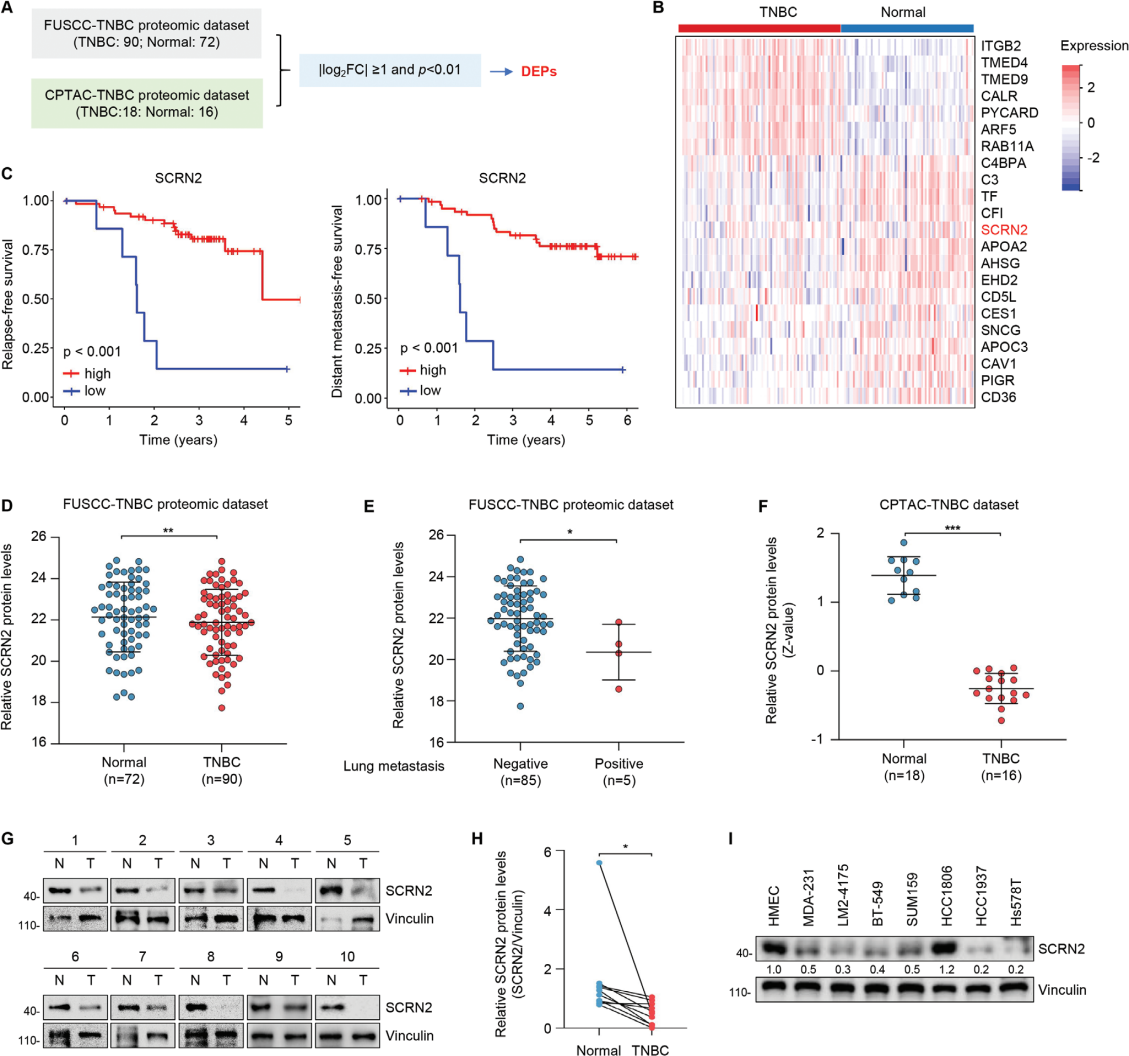
SCRN2 is downregulated in TNBC that is associated with poor patient prognosis. A) Cross‐analysis of the differentially expressed proteins (DEPs) between TNBC tissues and normal counterparts in TNBC proteomic datasets from the FUSCC and CPTAC. B) A total of 22 DEPs were obtained through the screening strategy described in panel A. C) Relapse‐free survival (RFS) and distant metastasis‐free survial (DMFS) of patients with high and low expression of SCRN2 in the FUSCC‐TNBC proteomic dataset. D) Analysis of the protein levels of SCRN2 in normal tissues and TNBC tissues in the FUSCC‐TNBC proteomic dataset. E) Analysis of the protein levels of SCRN2 in patients with or without lung metastasis in the FUSCC‐TNBC proteomic dataset. SCRN2 was detected in 4 of 5 paitents with lung metastasis.  F) Analysis of the protein levels of SCRN2 in normal tissues and TNBC tissues in the CPTAC‐TNBC proteomic dataset. G,H) Immunoblotting analysis of the protein expression levels of SCRN2 in 10 pairs of TNBC tissues and matched adjacent normal tissues (G) and the corresponding quantitative results (H). N, normal, T, tumor, n = 10. Pairwise *t*‐test was used to analyze the data in panel H. I) Immunoblotting analysis of the protein expression levels of SCRN2 in HMEC and 7 representative TNBC cell lines. The *p* values were calculated using the Student's *t‐*test between two groups. *p < 0.05, **p < 0.01, ***p < 0.001.

To identify the key genetic factors driving TNBC progression, we first analyzed the differentially expressed proteins between TNBC tissues and normal samples using TNBC proteomic datasets from our center (FUSCC)^[^ ^4^ ^]^ and the Clinical Proteomic Tumor Analysis Consortium (CPTAC)^[^ ^28^ ^]^ (**Figure** **1**A). Using this approach, a total of 22 differentially expressed proteins were identified according to the screening criteria of the absolute value of log_2_FC(fold change) over 1 and *p*‐value less than 0.01 in both datasets (Figure 1B). We next analyzed the relapse‐free survival (RFS) and distant metastasis‐free survival (DMFS) of TNBC patients with high and low expression of these proteins in the FUSCC‐TNBC proteomic dataset.^[^ ^4^ ^]^ The results showed that the expression levels of 12 screened proteins were associated with the prognosis of TNBC patients. Among them, low expression of SCRN2 was associated with poor RFS and DMFS of patients (Figure 1C). Of note, the roles of other 11 screened proteins in cancer progression have been documented previously, while the functional and mechanistic role of SCRN2 in human cancer remains unknown, Thus, we chose the poorly characterized SCRN2 for further investigation.

Next, we conducted a detailed analysis of SCRN2 protein expression in TNBC tissues and cell lines. In the FUSCC‐TNBC proteomics dataset, the expression levels of SCRN2 were downregulated in TNBC tissues relative to those in normal tissues (Figure 1D), and their expression levels were lower in TNBC patients with lung metastasis than in those without lung metastasis (Figure 1E). Similarly, we found that SCRN2 was expressed at low levels in TNBC tissues compared to normal tissues in the CPTAC dataset (Figure 1F). As the CPTAC dataset did not contain the information about the status of lung metastasis of patients, we could not analyze the correlation between the expression levels of SCRN2 with lung metastasis of patients in this dataset. To validate the above results, we collected 10 pairs of TNBC and adjacent normal tissues to examine the expression levels of SCRN2 by immunoblotting. Consistently, SCRN2 was significantly downregulated in TNBC tissues compared to normal counterparts (Figure 1G,H). We further assessed SCRN2 expression levels in a normal human mammary epithelial cell line HMEC and seven representative TNBC cell lines. Results showed that the SCRN2 expression levels were generally lower in most tested TNBC cell lines (except HCC1806) than those in HMEC (Figure 1I). Taken together, these results indicate that SCRN2 is downregulated in TNBC, and that its downregulation is associated with poor patient prognosis.

In addition to SCRN2, the secernin family includes SCRN1 and SCRN3.^[^ ^5^, ^6^ ^]^ Thus, we also analyzed the expression of SCRN1 and SCRN3 in TNBC. In the FUSCC‐TNBC proteomic dataset, the expression levels of SCRN1 were relatively higher in TNBC tissues than in normal samples (Figure S1A, Supporting Information), however, there was no difference in expression levels between patients with or without lung metastasis (Figure S1B, Supporting Information). In addition, no differences were observed in the expression levels of SCRN1 between normal and TNBC tissues in the CPTAC dataset (Figure S1C, Supporting Information). For SCRN3, the median expression levels were higher in TNBC tissues than in normal tissues in the FUSCC‐TNBC proteomic dataset. However, we could not perform statistical analysis because of the low detection rate in normal tissues (Figure S1D, Supporting Information). Similarly, we could not perform statistical analysis for the expression levels of SCRN3 in patients with or without lung metastasis, as SCRN3 was not detected in patients with lung metastasis (Figure S1E, Supporting Information). In the CPTAC dataset, no significant differences were observed in the expression levels of SCRN3 between normal and TNBC tissues (Figure S1F, Supporting Information). These results suggest that among the members of the secernin family, SCRN2 is specifically downregulated in TNBC and may function as a tumor suppressor.

### 2.2 Genetic Alternations and Promoter Methylation do not Significantly Contribute to SCRN2 Downregulation in TNBC

Accumulating evidence shows that the downregulation of tumor suppressor genes is achieved through three main mechanisms, including genetic alterations, promoter methylation, or protein instability.^[^ ^29^, ^30^ ^]^ To address the underlying mechanism of SCRN2 downregulation in TNBC, we first analyzed genetic alterations of SCRN2 using the cBioPortal for Cancer Genomics (https://www.cbioportal.org).^[^ ^31^ ^]^ Results showed that the mutation frequency of SCRN2 in all breast cancers and TNBC was lower than 1% (Figure S2A–C, Supporting Information). In addition, the frequency of copy number deletion of SCRN2 in all breast cancers and TNBC was ≈ 0.5% and 0%, respectively (Figure S3A–C, Supporting Information). Thus, genetic alterations were not primarily involved in SCRN2 downregulation in TNBC.

DNA methylation is a general epigenetic mechanism involved in gene silencing in human cancer.^[^ ^29^ ^]^ To examine whether abnormal epigenetic methylation contributes to SCRN2 downregulation in TNBC, we first analyzed the CpG islands of SCRN2 promoter (from ‐1000 bp to +100 bp relative to transcription start site) using the MethPrimer program (http://www.urogene.org/index.html).^[^ ^32^ ^]^ Following the established criteria of CpG island size > 100 bp, GC percentage > 50%, and observed/expected CpG ratio > 0.6,^[^ ^33^ ^]^ we detected a CpG island in SCRN2 promoter (Figure S4A, Supporting Information). Next, we examined the methylation levels of SCRN2 promoter in 23 breast cancer cell lines using Cancer Cell Line Encyclopedia (CCLE) dataset (https://www.broadinstitute.org/ccle/home). Results showed that SCRN2 promoter generally possessed low DNA methylation levels in most breast cancer cell lines, including TNBC cells (Figure S4B, Supporting Information). We next used UCSC Xena (https://xena.ucsc.edu)^[^ ^34^ ^]^ to analyze SCRN2 promoter methylation status in TNBC. Results showed that the methylation levels of SCRN2 promoter in TNBC tissues were slightly higher than those in normal tissues (Figure S4C, Supporting Information). To further validate the above results, we treated cells with DNA methylation inhibitor 5‐aza‐2′ ‐deoxycytidine (5‐aza‐dC) and then detected the change of SCRN2 expression levels. Results showed that 5‐aza‐dC treatment did not significantly alter the protein levels of SCRN2 (Figure S4D, Supporting Information). Taken together, these results indicate that promoter methylation is not the main mechanism underlying SCRN2 downregulation in TNBC cells.

### 2.3 SCRN2 is Degraded through Chaperone‐Mediated Autophagy (CMA) Pathway in TNBC

**Figure 2:**
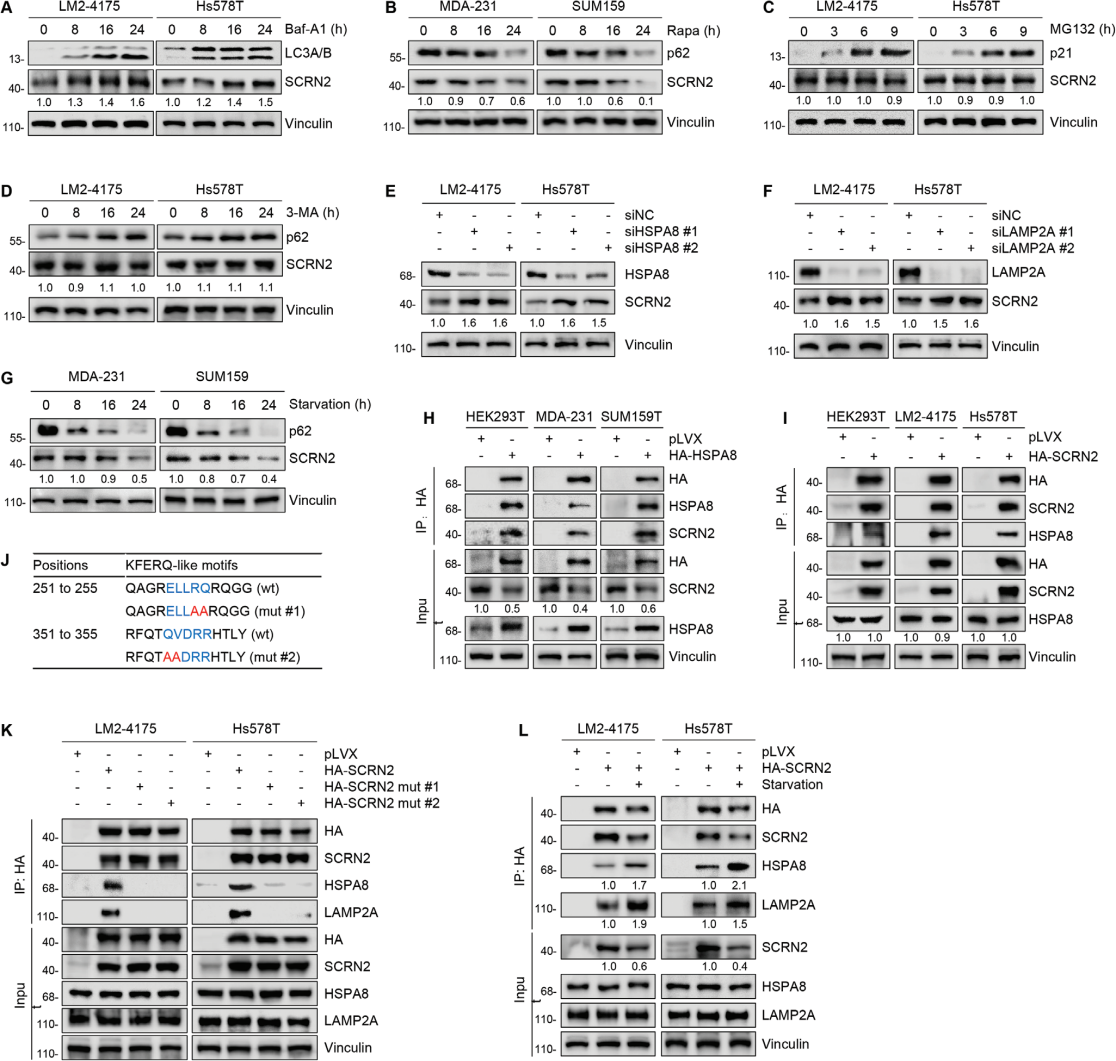
SCRN2 is degraded through chaperone‐mediated autophagy pathway in TNBC. A‐D) Changes of the protein expression levels of SCRN2 after Baf‐A1 (A), rapamycin (B), MG132 (C), and 3‐MA (D) treatment. E,F) Immunoblotting analysis of SCRN2 protein expression levels following knockdown of HSPA8 (E) or LAMP2A (F) using two siRNAs. G) Immunoblotting analysis of SCRN2 protein expression levels in cells after serum starvation for the indicated times. H,I) HEK293T and TNBC cells were transfected with the indicated expression vectors and then subjected to IP assays to detect the interaction of HSPA8 with SCRN2. J) Analysis of the KFERQ‐like motifs in SCRN2 protein using the KFERQ motif finder program (https://rshine.einsteinmed.edu). The generation of the mutated KFERQ‐like motifs is shown below its wild‐type (wt) counterpart. K) Cells were transfected with the indicated expression vectors and then subjected to IP assays to detect the interaction of SCRN2 (wt and KFERQ motif‐mutant) with HSPA8 and LAMP2A. L) Cells expressing empty vector pLVX and HA‐SCRN2 were treated with or without serum starvation for 24 h and then subjected to IP assays to detect the interaction of SCRN2 with HSPA8 and LAMP2A.

The ubiquitin‐proteasome and autophagy‐lysosome pathways are the two main routes of protein degradation in eukaryotes.^[^ ^35^ ^]^ To investigate whether protein instability could be the cause of SCRN2 downregulation in TNBC, we treated TNBC cells with lysosome inhibitor bafilomycin‐A1 (Baf‐A1), autophagy inducer rapamycin (Rapa), and proteosome inhibitor MG132 and then examined the change of SCRN2 protein levels. Results showed that the protein levels of SCRN2 were increased after Baf‐A1 treatment, whereas were decreased following rapamycin treatment (**Figure** **2**A,B). In contrast, there were no obvious changes in the protein levels of SCRN2 in response to MG132 treatment (Figure 2C). These results indicate that SCRN2 is degraded mainly through the autophagy‐lysosome pathway rather than the ubiquitin‐proteasome pathway.

Macroautophagy, microautophagy, and chaperone‐mediated autophagy (CMA) are the three main types of autophagy.^[^ ^36^, ^37^ ^]^ Macroautophagy delivers various cellular contents to lysosomes for degradation, whereas microautophagy is still poorly understood in mammals.^[^ ^36^ ^]^ In contrast, CMA is responsible for selective degradation of proteins bearing a Lys‐Phe‐Glu‐Arg‐Gln (KFERQ)‐like motif.^[^ ^37^ ^]^ This motif is selectively recognized by the chaperone protein HSPA8 (heat shock protein family A member 8), which transfers substrates to lysosomes for degradation by interacting with LAMP2A (lysosomal associated membrane protein 2A).^[^ ^37^ ^]^ To explore which types of autophagy are involved in SCRN2 degradation in TNBC, we first treated LM2‐4175 and Hs578T cells with classic macroautophagy inhibitor 3‐methyladenine (3‐MA),^[^ ^38^ ^]^ but found no obvious changes in the protein levels of SCRN2 after 3‐MA treatment (Figure 2D). This result indicates that SCRN2 may not be degraded by macroautophagy. To determine whether SCRN2 is degraded through the CMA pathway, we knocked down HSPA8 and LAMP2A, two core components of the CMA machinery,^[^ ^37^ ^]^ using two specific siRNAs, and found an increase in SCRN2 protein levels following the depletion of either HSPA8 or LAMP2A (Figure 2E,F). In line with these observations, the induction of CMA through prolonged nutrient starvation also resulted in the upregulation of SCRN2 protein levels (Figure 2G). Reciprocal IP assays further demonstrated that SCRN2 interacted with HSPA8 (Figure 2H,I), which is the only chaperone protein that directly binds to the KFERQ‐like motif in the CMA substrates.^[^ ^37^ ^]^ Consistently, we found that SCRN2 protein contains two KFERQ‐like motifs (Figure 2J). Furthermore, the mutation of one of the two KFERQ‐like motifs in SCRN2 significantly attenuated the interaction of SCRN2 with HSPA8 and LAMP2A (Figure 2K), whereas the induction of CMA through nutrient starvation increased their interactions (Figure 2L). Taken together, these results suggest that SCRN2 is a bona fide CMA substrate that is degraded by CMA in TNBC cells.

### 2.4 SCRN2 Suppresses the Proliferative, Migratory, and Invasive Potential of TNBC Cells

**Figure 3:**
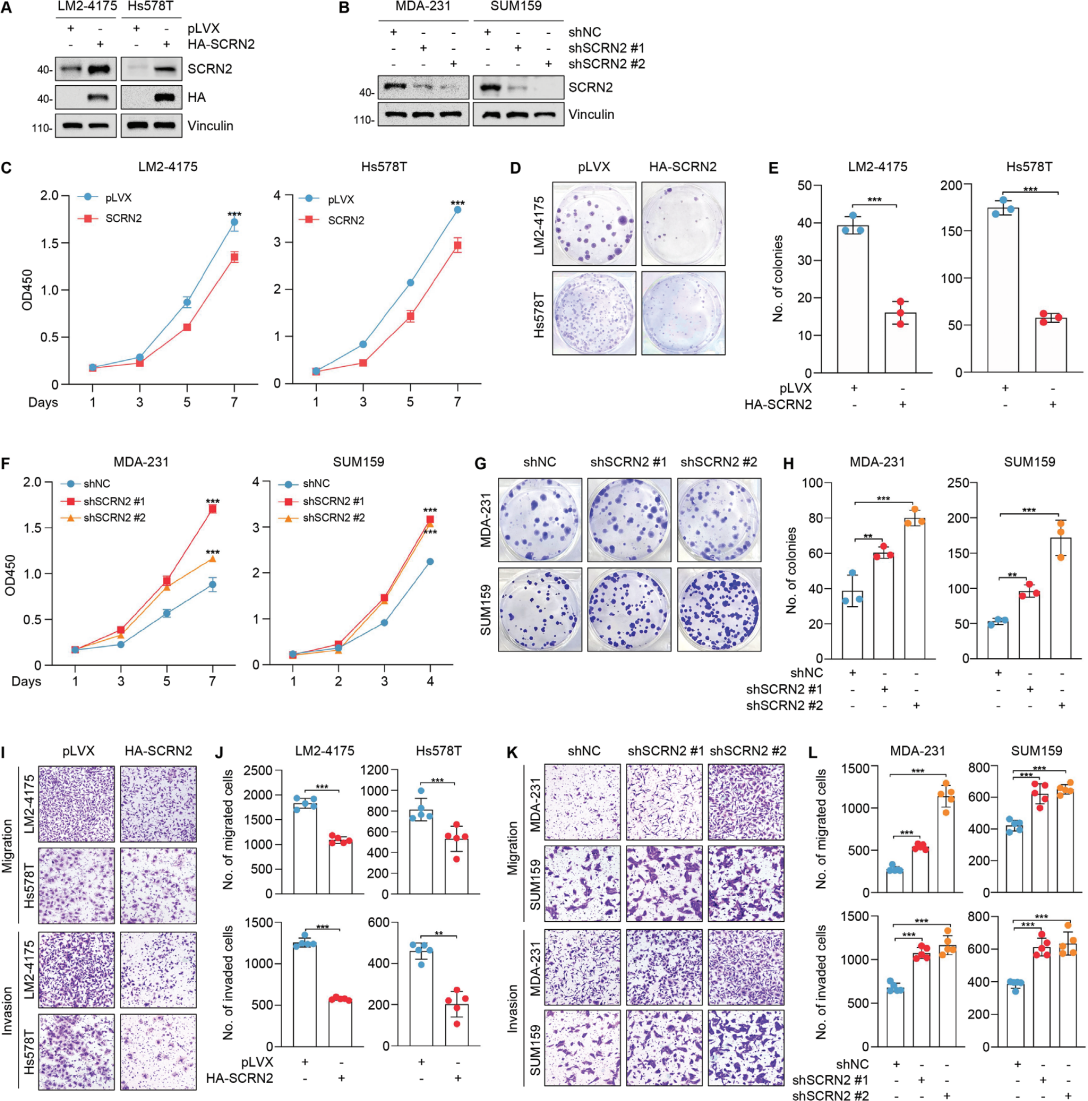
SCRN2 suppresses the proliferative, migratory, and invasive potential of TNBC cells. A,B) Immunoblotting validation of expression status of SCRN2 in the established stable cell lines expressing HA‐SCRN2 (A) or shRNAs targeting SCRN2 (B). C‐E) LM2‐4175 and Hs578T cells stably expressing pLVX and HA‐SCRN2 were subjected to CCK‐8 (n = 5) (C) and colony formation assays (n = 3) (D and E). The representative images of survival colonies and the corresponding quantitative results are shown in D and E, respectively. F‐H) MDA‐MB‐231 and SUM159 cells stably expressing shNC and shSCRN2 were subjected to CCK‐8 (n = 5) (F) and colony formation assays (n = 3) (G and H). The representative images of survival colonies and the corresponding quantitative results are shown in G and H, respectively. I,J) LM2‐4175 and Hs578T cells stably expressing pLVX and HA‐SCRN2 were subjected to Transwell migration and invasion assays. The representative images of the migrated and invaded cells and the corresponding quantitative results are shown in I and J, respectively. n = 5 per group. K,L) MDA‐MB‐231 and SUM159 cells stably expressing shNC and shSCRN2 were subjected to Transwell migration and invasion assays. The representative images of the migrated and invaded cells and the corresponding quantitative results are shown in K and L, respectively. n = 5 per group. The *p* values were calculated using the Student's *t‐*test between two groups. *p < 0.05, **p < 0.01, ***p < 0.001.

Based on the expression levels of SCRN2 in TNBC cell lines (Figure 1I), we stably overexpressed HA‐SCRN2 in LM2‐4175 and Hs578T cells (**Figure** **3**A), and depleted endogenous SCRN2 in MDA‐MB‐231 and SUM159 cells using two independent shRNAs targeting SCRN2 (shSCRN2 #1 and #2) (Figure 3B) for functional assays. CCK‐8 and colony formation assays demonstrated that overexpression of SCRN2 decelerated the proliferative potential and colony formation capacity of LM2‐4175 and Hs578T cells (Figure 3C–E), while opposite results were obtained when SCRN2 was knocked down in MDA‐MB‐231 and SUM159 cells (Figure 3F–H). Transwell migration and invasion assays revealed that overexpression of SCRN2 suppressed, whereas knockdown of SCRN2 promoted, the migratory and invasive abilities of TNBC cells (Figure 3I,L). Taken together, these results suggest that SCRN2 acts as a tumor suppressor in TNBC.

### 2.5 SCRN2 Stabilizes KMT2C by Impeding its Proteasomal Degradation

**Figure 4:**
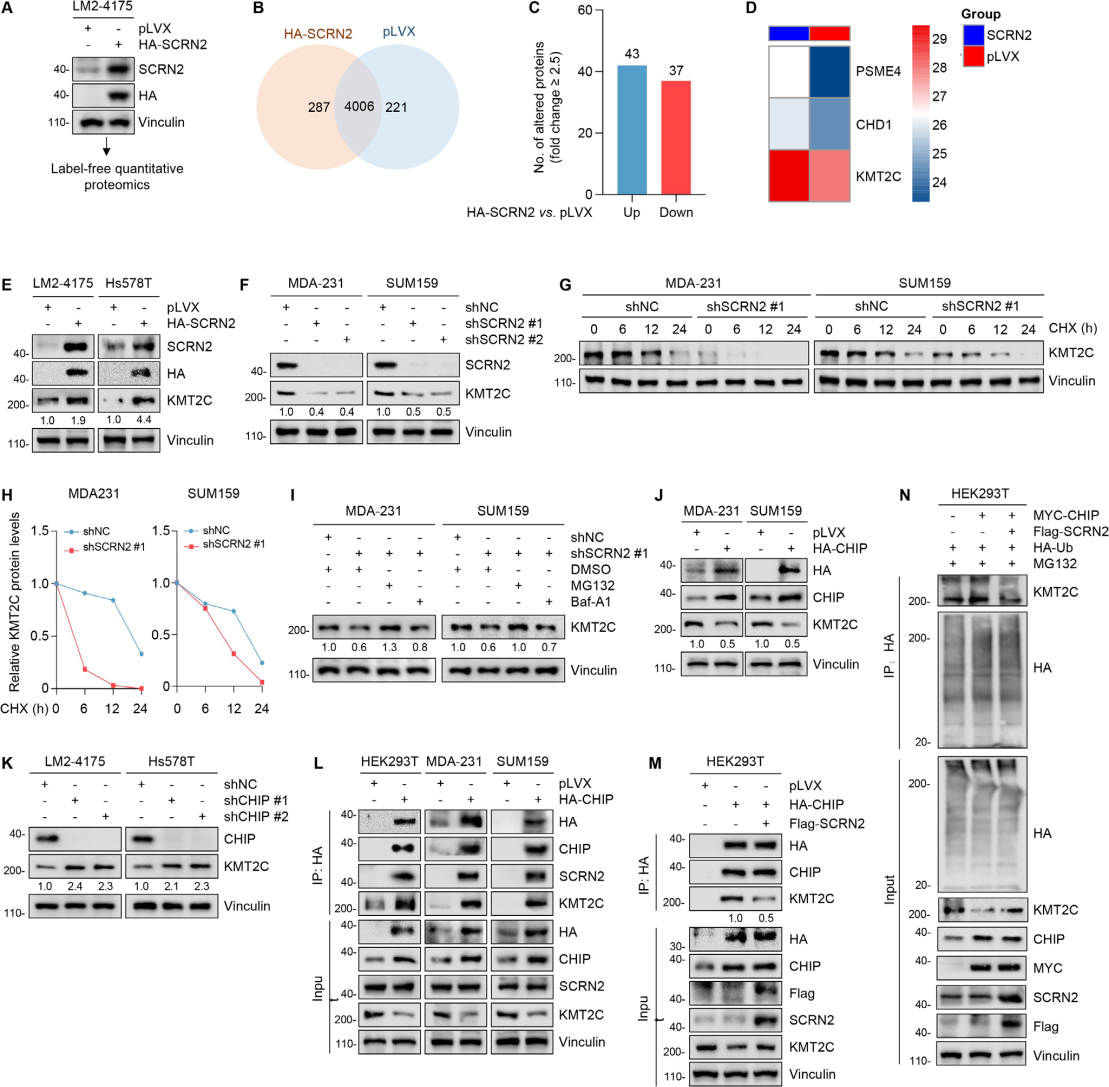
SCRN2 impedes proteasomal degradation of KMT2C by blocking its interaction with E3 ubiquitin‐protein ligase CHIP. A) LM2‐4175 cells stably expressing SCRN2 were subjected to label‐free quantitative proteomic assays. B) The number of the identified proteins by mass spectrum in LM2‐4175 cells stably expressing pLVX and HA‐SCRN2. C) A total of 80 differentially expressed proteins were obtained according to fold change ≥2.5. Of them, 43 proteins were upregulated while 37 were downregulated after SCRN2 overexpression. D) Three differentially expressed proteins that are associated with chromosome or chromatin organization were obtained after cross‐analysis of the results of biological process and molecular function in GO analyses. E,F) Immunoblotting analysis of the expression levels of KMT2C in SCRN2‐overexpressing (E) and ‐depleted (F) cells. G,H) Cells expressing shNC or shSCRN2 were subjected to CHX chase assays. The representative images of immunoblotting and the corresponding quantitative results are shown in G and H, respectively. I) Cells expressing shNC or shSCRN2 were treated with or without DMSO, MG132, and Baf‐A1, and subjected to immunoblotting analysis of the expression levels of KMT2C. J,K) Immunoblotting analysis of the expression levels of KMT2C in CHIP‐overexpressing (J) and ‐depleted (K) TNBC cells. L) Cells were transfected with the indicated expression vectors and subjected to IP assays to detect the interaction of CHIP with KMT2C. M) Cells were transfected with the indicated expression vectors and subjected to IP assays to detect the effects of SCRN2 on the interaction of CHIP with KMT2C. N) Cells were transfected with the indicated expression vectors and subjected to IP assays to detect the effects of CHIP and SCRN2 on KMT2C ubiquitination.

To reveal the molecular mechanisms underlying SCRN2 suppression of TNBC progression, we performed label‐free quantitative proteomic assays using SCRN2‐overexpressing LM2‐4175 cells to identify the regulatory proteins (**Figure** **4**A,B). According to the selection criteria of fold change greater than or equal to 2.5, 80 differentially expressed proteins were identified, of which 43 were upregulated and 37 were downregulated after SCRN2 overexpression (Figure 4C). To further understand the roles of these differentially expressed proteins, we performed comprehensive Gene Ontology (GO) analysis. According to the classification of biological processes, these proteins participate in multiple cancer‐related pathways, such as apoptotic processes, chromosome/chromatin organization, and response to DNA damage stimuli (Figure S5A, Supporting Information). Additionally, molecular function analysis indicated that these proteins have RNA‐binding activity, transcriptional co‐regulator activity, and histone‐binding activities (Figure S5B, Supporting Information). In terms of cellular components, these proteins contributed to supramolecular complexes, spindles, and chromosomal regions (Figure S5C, Supporting Information).

The packaging of DNA into chromatin in eukaryotes regulates all DNA‐templated reactions, including gene transcription and the DNA damage response.^[^ ^39^ ^]^ Consequently, the disruption of chromatin organization has profound effects on these vital biological processes. Cross‐analysis of the results of biological processes and molecular functions in GO analyses revealed that three differentially expressed proteins, including proteasome activator complex subunit 4 (PSME4, chromodomain‐helicase‐DNA‐binding protein 1 (CHD1), and KMT2C, were associated with chromosome or chromatin organization (Figure 4D). Prognostic analysis revealed that only KMT2C was an independent prognostic marker for TNBC (Figure S6A–C, Supporting Information). Therefore, KMT2C was selected for further validation.

Next, we determined whether SCRN2 influenced the expression levels of KMT2C. Results showed that SCRN2 upregulated KMT2C expression at the protein level but not at the mRNA level (Figure 4E,F; Figure S7A,B, Supporting Information). Cycloheximide (CHX) chase assays showed that knockdown of SCRN2 shortened the half‐life of KMT2C (Figure 4G,H), indicating that SCRN2 enhances the stability of KMT2C. Furthermore, treatment of cells with the proteasome inhibitor MG132, but not lysosome inhibitor Baf‐A1, resulted in increased KMT2C protein levels in a time‐dependent manner (Figure S7C,D, Supporting Information). Importantly, the downregulation of KMT2C caused by SCRN2 knockdown was partially restored following incubation of cells with MG132, but not with Baf‐A1 (Figure 4I). Collectively, these results suggest that SCRN2 stabilizes KMT2C by impeding its proteasomal degradation.

### 2.6 SCRN2 Blocks the Interaction of KMT2C with E3 Ubiquitin‐Protein Ligase CHIP to Suppress KMT2C Ubiquitination

As SCRN2 is not a putative deubiquitinase, we speculated that SCRN2 stabilizes KMT2C probably through other regulators. To explore this possibility, we transfected pLVX and HA‐SCRN2 vectors into HEK293T cells and performed liquid chromatography‐tandem mass spectrometry (LC‐MS/MS) assays to identify potential binding partners of SCRN2. By this means, a total of 52 proteins were identified according to the screening criteria of the identified unique peptides greater than 2. GO analysis revealed that these proteins were associated with RNA metabolic processes, regulation of protein stability, and transmembrane transport (Figure S8A, Supporting Information). Based on the number and coverage of the unique peptides identified, we ranked the binding partners of SCRN2 that were enriched in the pathway regulating protein stability (Figure S8B, Supporting Information). HSPA8 was also one of the binding partners enriched in this pathway, further confirming that HSPA8 mediates SCRN2 degradation. Another interacting protein of SCRN2 is the BAG family molecular chaperone regulator 2 (BAG2), which belongs to the Bcl2‐associated athanogene (BAG) protein family and interferes with the interaction of the E3 ubiquitin‐protein ligase CHIP (also known as STUB1) with its substrates, thus suppressing the CHIP‐mediated ubiquitination of its substrates.^[^ ^40^, ^41^ ^]^ In addition, using the UbiBrowser version 2.0 database (http://ubibrowser.ncpsb.org.cn), which is a comprehensive resource for proteome‐wide known and predicted ubiquitin ligase/deubiquitinase‐substrate interactions in eukaryotic species,^[^ ^42^ ^]^ we predicted that CHIP is a potential E3 ligase for KMT2C, as 6 recognition motifs of CHIP were presented within the KMT2C amino acid sequence with a high level of confidence (confidence score = 0.809) (Figure S8C,D, Supporting Information). Therefore, we hypothesized that SCRN2 might inhibit the ubiquitination of KMT2C by recruiting BAG2 to interfere with the interaction between CHIP and KMT2C. To confirm this hypothesis, we performed IP assays and found that SCRN2 interacted with BAG2 (Figure S8E–G, Supporting Information). Additionally, BAG2 positively regulated the expression of KMT2C at the protein level but not at the mRNA level (Figure S8H,I, Supporting Information). Next, we examined whether CHIP is an E3‐ligase of KMT2C. As shown in Figure 4J,K, Figure S8J,K (Supporting Information), overexpression of CHIP decreased the protein levels of KMT2C, whereas knockdown of CHIP yielded the opposite results. Moreover, these changes were not observed at the mRNA level. In addition, CHIP interacted with KMT2C and promoted its ubiquitination, while whereas the expression of SCRN2 interfered with this interaction and reduced the ubiquitination of KMT2C (Figure 4L–N). Taken together, these results indicate that SCRN2 stabilizes KMT2C by impeding the interaction between CHIP and KMT2C, thereby suppressing ubiquitin‐dependent proteasomal degradation of KMT2C.

### 2.7 SCRN2 Inhibits TNBC Growth and Metastasis Partially through KMT2C both in Vitro and In Vivo

**Figure 5:**
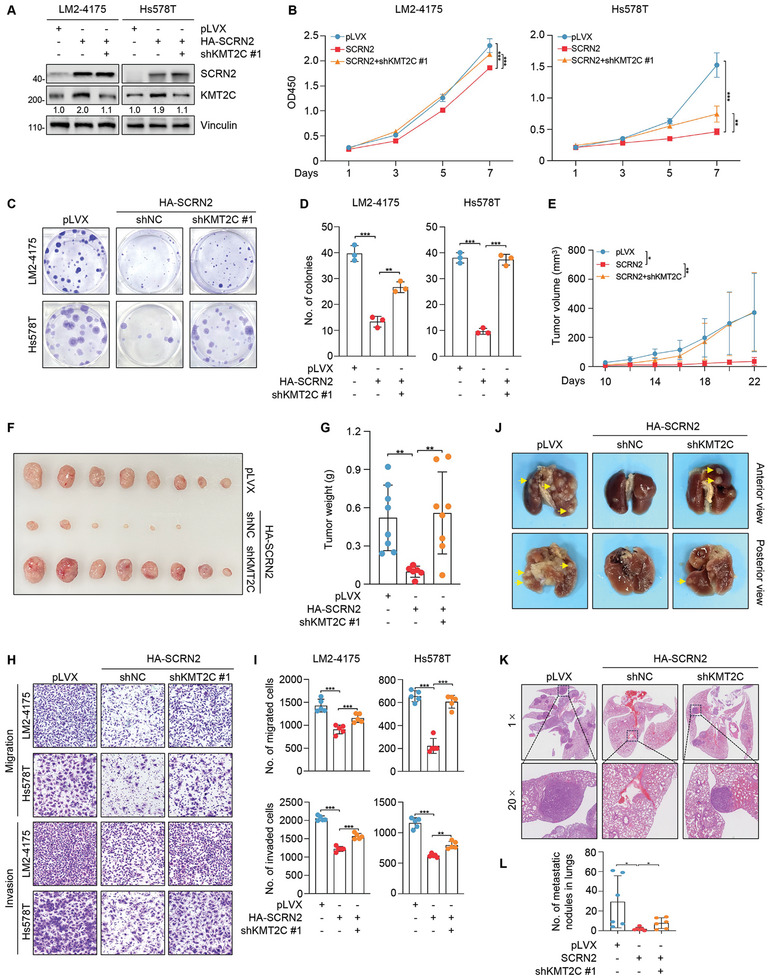
SCRN2 suppresses TNBC progression partially through KMT2C both in vitro and in vivo. A) Cells expressing pLVX, HA‐SCRN2 alone or in combination with shKMT2C were subjected to immunoblotting analysis to validate the established stable cell lines. B‐D) Cells expressing pLVX, HA‐SCRN2 alone or in combination with shKMT2C were subjected to CCK‐8 (n = 5) (B) and colony formation assays (n = 3) (C and D). The representative images of survival colonies and the corresponding quantitative results are shown in C and D, respectively. E‐G) Cells expressing pLVX, HA‐SCRN2 alone or in combination with shKMT2C were injected into the mammary fat pad of 6‐week‐old BALB/c female nude mice. After 22 days of injection, mice were sacrificed and xenograft tumors were removed. Growth curve (E), representative image (F), and weight (G) of xenograft tumors are shown. n = 8 per group. Two mice in the HA‐SCRN2 group died of natural causes before the end of the experiment. H,I) Cells expressing pLVX, HA‐SCRN2 alone or in combination with shKMT2C were subjected to Transwell migration and invasion assays. Representative images of the migrated and invaded cells (H) and the corresponding quantitative results (I) are shown. n = 5 per group. J‐L) Cells expressing pLVX, HA‐SCRN2 alone or in combination with shKMT2C were injected into the tail vein of 6‐week‐old BALB/c female nude mice. After about 60 days of injection, mice were sacrificed and lungs were removed and subjected to HE staining. Representative images of lungs (J), HE‐staining (K) and the quantitative results of lung metastatic nodules (L) are shown. n = 6 per group. The *p* values were calculated using the Student's *t‐*test between two groups. *p < 0.05, **p < 0.01, ***p < 0.001.

To address whether SCRN2 inhibited TNBC progression through KMT2C, we knocked down KMT2C in SCRN2‐overexpressing cells (**Figure** **5**A) and performed in vitro and in vivo functional assays. CCK8 and colony formation assays showed that knockdown of KMT2C partially rescued the decreased proliferative potential, colony formation capacity, and xenograft tumor growth in mice caused by SCRN2 overexpression (Figure 5B–G).

To assess whether SCRN2 inhibits TNBC cell migration, invasion, and metastasis through KMT2C, we carried out transwell migration and invasion assays and experimental lung metastasis assays by injecting cells into the tail veins of BALB/c female nude mice using established cell lines. As shown in Figure 5H, knockdown of KMT2C reversed the impaired migratory and invasive abilities of cells caused by SCRN2 overexpression. Furthermore, SCRN2 partially inhibited TNBC metastasis through KMT2C in vivo (Figure 5I–L; Figure S9, Supporting Information). Taken together, SCRN2 partially inhibits TNBC growth and metastasis through KMT2C both in vitro and in vivo.

### 2.8 SCRN2 Epigenetically Regulates BMF and Promotes Intrinsic Apoptosis through KMT2C

**Figure 6:**
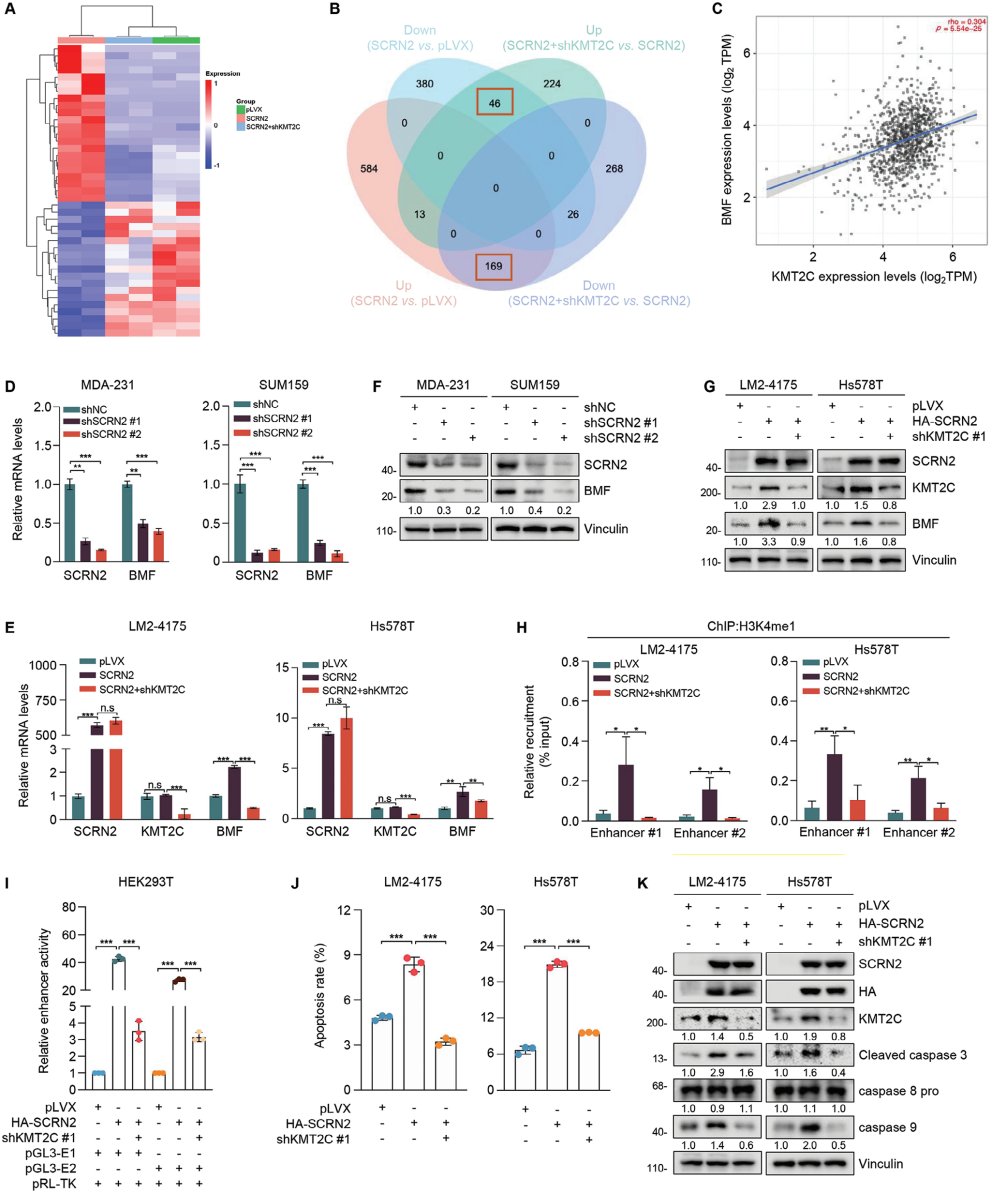
SCRN2 transcriptionally activates pro‐apoptotic factor BMF by amplifying H3K4me1 at its enhancer via KMT2C. A,B) LM2‐4175 cells expressing pLVX, HA‐SCRN2 alone or in combination with shKMT2C were subjected to RNA‐Seq assays, each group had two replicates. Heatmap (A) and the Venn map (B) of the differentially expressed genes are shown. The selection criteria of the differentially expressed genes was *p*‐value less than 0.05 and the absolute value of log_2_FC (fold change) over 1. C) Correlation of the expression levels of KMT2C and BMF in the TCGA dataset. D,E) qPCR analysis of BMF mRNA levels in cells expressing shSCRN2 (D) and in cells expressing pLVX, HA‐SCRN2 alone or in combination with shKMT2C (E). F,G) Immunoblotting analysis of BMF protein levels in cells expressing shSCRN2 (F) and in cells expressing pLVX, HA‐SCRN2 alone or in combination with shKMT2C (G). H) Detection of the H3K4me1 levels at BMF enhancers by ChIP‐qPCR assays. I) Detection of BMF enhancer activities by dual‐luciferase reporter assays in cells expressing pLVX, HA‐SCRN2 alone or in combination with shKMT2C. J) Detection of apoptosis by flow cytometry assays in cells expressing pLVX, HA‐SCRN2 alone or in combination with shKMT2C. Each group had 3 replicates. K) Immunoblotting analysis of the activation of extrinsic and intrinsic apoptosis pathway in cells expressing pLVX, HA‐SCRN2 alone or in combination with shKMT2C. *p* values were calculated using the Student's *t‐*test between different group. *p < 0.05, **p < 0.01, ***p < 0.001.

KMT2C belongs to the KMTs family and mainly participates in H3K4me1 modification of the enhancers of its downstream target genes.^[^ ^21^, ^43^, ^44^ ^]^ To identify downstream target genes that are concurrently regulated by SCRN2 and KMT2C, we conducted RNA‐sequencing (RNA‐Seq) using cells expressing the empty vector, HA‐SCRN2 alone or in combination with KMT2C knockdown (**Figure** **6**A). Following the selection criteria of a *p*‐value less than 0.05 and an absolute value of log_2_FC (fold change) over 1, a total of 215 differentially expressed genes were obtained (Figure 6B). Next, we performed a GO analysis of the differentially expressed genes. In the biological process category, these genes were associated with apoptotic process, cell migration, and cell activation (Figure S10A, Supporting Information). In addition to the apoptotic process, the differentially expressed genes were enriched in two other apoptosis‐related pathways (Figure S10B, Supporting Information). Cross‐analyses of the three apoptosis‐related pathways revealed 8 apoptosis‐associated genes (Figure S10C, Supporting Information). Among them, the pro‐apoptotic factor BMF (Bcl2‐modifying factor) was chosen for further validation because its expression levels showed the highest correlation with those of KMT2C (Figure 6C, Figure S10D–J, Supporting Information).

BMF belongs to the proapoptotic BH3‐only protein subgroup of the BCL‐2 family and was initially described to sense cell stress and induce rapid Bcl2‐blockable apoptosis.^[^ ^45^ ^]^ Under multiple stress signals, BMF is highly regulated at both the transcriptional and post‐transcriptional levels.^[^ ^46^ ^]^ To validate whether SCRN2 and KMT2C regulate BMF in TNBC, we first examined changes in BMF expression when SCRN2 and KMT2C were genetically manipulated. As expected, SCRN2 positively regulated the expression of BMF both at the mRNA and the protein levels, and this process was mediated by KMT2C (Figure 6D–G). As KMT2C epigenetically regulates gene expression mainly by promoting H3K4me1 modification of target gene enhancers, we investigated whether SCRN2 stimulates BMF expression through KMT2C‐mediated H3K4me1 modification of its enhancer. Immunoblotting assays demonstrated that SCRN2 positively modified the whole H3K4me1 levels in cells and that this modification was partially depended on KMT2C (Figure S11, Supporting Information). Then, we designed two different primers for BMF enhancers according to the UCSC Genome Browser (https://genome.ucsc.edu/) and performed ChIP‐qPCR. As shown in Figure 6H, H3K4me1 levels on both BMF enhancers increased when SCRN2 was overexpressed, whereas they decreased following the knockdown of KMT2C. Moreover, dual‐luciferase reporter assays demonstrated that the activity of these two enhancer regions increased following SCRN2 overexpression and decreased after KMT2C knockdown (Figure 6I). These results indicate that SCRN2 enhances H3K4me1 modification of the BMF gene enhancer, thereby promoting BMF transcription through KMT2C.

As BMF is mainly involved in the regulation of apoptosis, we investigated whether SCRN2 and KMT2C participated in apoptosis. Flow cytometry assays showed that the apoptotic rate increased when SCRN2 was overexpressed, whereas it decreased after KMT2C knockdown (Figure 6J; Figure S12, Supporting Information), indicating that SCRN2 promotes apoptosis through KMT2C. Apoptosis can be triggered by death receptor ligation on the cell surface and cellular stress in the mitochondria via the extrinsic and intrinsic apoptotic pathways, respectively. Additionally, the intrinsic apoptotic pathway is mainly regulated by the Bcl‐2 protein family.^[^ ^47^ ^]^ To distinguish the apoptotic pathways in which SCRN2 is mainly involved, we performed immunoblotting assays to detect the levels of cleaved‐caspase 3, caspase 8 pro and caspase 9, which are markers of general apoptosis, extrinsic pathway, and intrinsic pathway, respectively. The results showed that SCRN2 and KMT2C did not alter the levels of caspase 8 pro, but positively regulated the levels of cleaved‐caspase 3 and caspase 9 (Figure 6K; Figure S13, Supporting Information), suggesting that SCRN2 and KMT2C promoted the intrinsic apoptotic pathway in TNBC cells.

To further confirm the HSPA8/SCRN2/KMT2C/BMF axis in TNBC cells, we generated cell lines with either overexpression or knockdown of HSPA8 and conducted immunoblotting assays. Results showed that SCRN2, KMT2C, BMF and the whole H3K4me1 levels were downregulated in HSPA8‐overexpressing cells, but upregulated in HSPA8‐knocked down cells (Figure S14, Supporting Information), confirming the function of the HSPA8/SCRN2/KMT2C/BMF axis in TNBC cells. Collectively, these results suggest that SCRN2 promotes H3K4me1 levels in BMF enhancers to induce BMF expression and activate the intrinsic apoptotic pathway through KMT2C in TNBC cells.

### 2.9 SCRN2 Induces Endogenous DNA Damage and Sensitizes TNBC cells to PARP Inhibition

**Figure 7:**
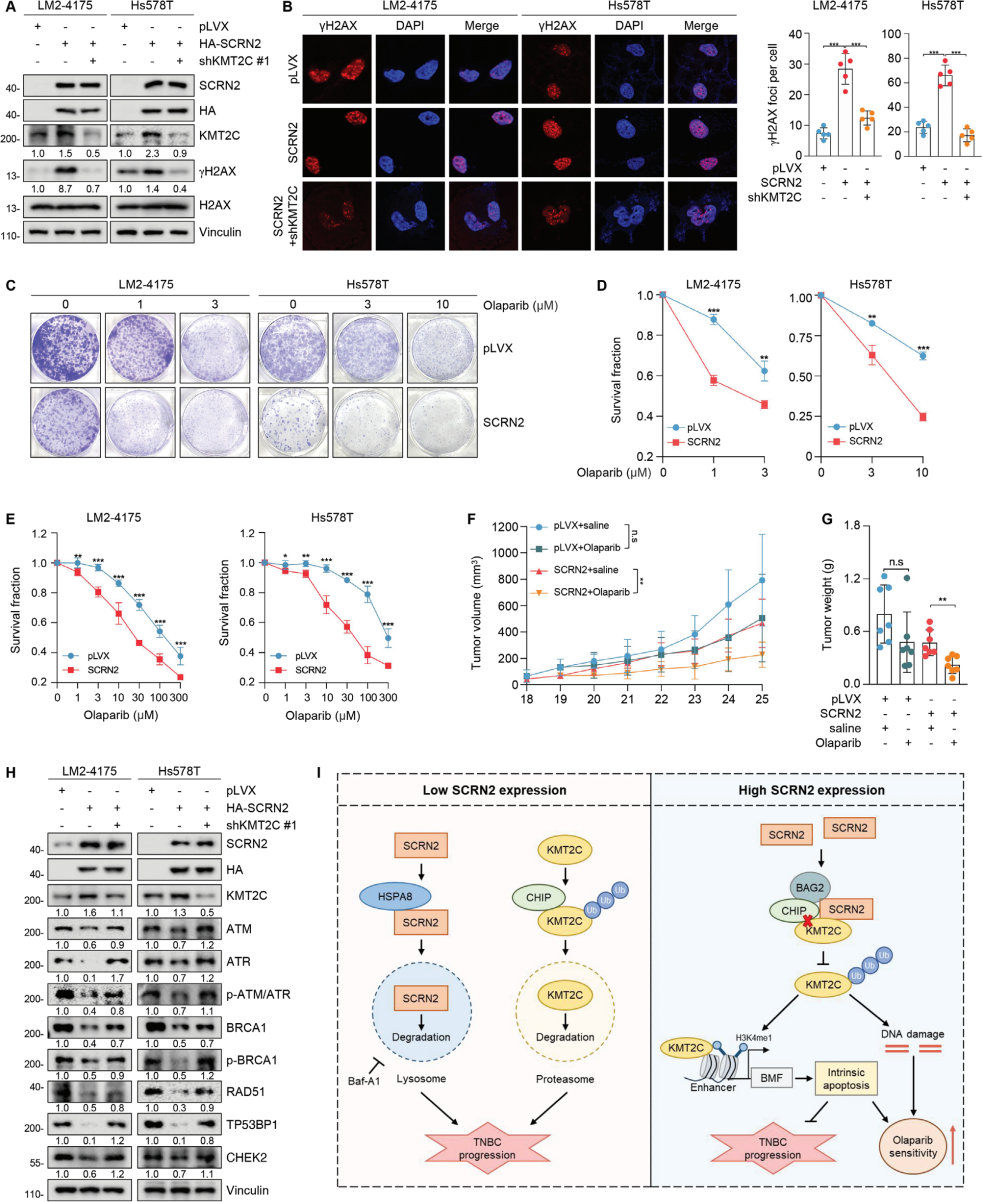
SCRN2 triggers endogenous DNA damage and sensitizes TNBC cells to Olaparib. A,B) DNA damage status in cells expressing pLVX, HA‐SCRN2 alone or in combination with shKMT2C was detected by immunoblotting (A) and immunofluorescent staining (B). γ‐H2AX was used as a marker of DNA damage. C,D) Cells expressing pLVX and HA‐SCRN2 were treated with or without different doses of Olaparib and subjected to colony formation assays. Representative images of survival colonies (C) and the corresponding quantitative results (D) are shown. n = 3 per group. E) Cells expressing pLVX and HA‐SCRN2 were treated with or without different doses of Olaparib and subjected to CCK8 assays to detect cell viability. Survival fraction was calculated. n = 5 per group. F,G) LM2‐4175 cells expressing pLVX and HA‐SCRN2 were injected into the fat pad of 6‐weeks‐old BALB/c female nude mice. When the tumor volume of one of the groups reached 100 mm^3^, mice were treated with saline or 50 mg kg^−1^ Olaparib through oral gavage for 7 times. Tumor volume was measured every day. The growth curve (F) and weight (G) of xenograft tumors are shown. H) Immunoblotting analysis of the expression and activation of DNA damage repair‐related genes in cells expressing pLVX, HA‐SCRN2 alone or in combination with shKMT2C. I) The proposed working model. SCRN2 is degraded in TNBC cells through HSPA8‐mediated CMA pathway, resulting in SCRN2 downregulation and its inability to stabilize KMT2C to promote TNBC progression (left panel). While SCRN2 could not be degraded by HSPA8, SCRN2 recruits BAG2 to interfere the interaction of CHIP with KMT2C to stabilize KMT2C and increase the H3K4me1 levels at BMF enhancers, therefore inducing intrinsic apoptosis and inhibiting TNBC progression. In addition, SCRN2 triggers endogenous DNA damage via KMT2C and sensitizes TNBC cells to Olaparib. The *p* values were calculated using the Student's *t‐*test between two groups. *p < 0.05, **p < 0.01, ***p < 0.001, n.s, not significant.

KMT2C has been reported to influence the therapeutic effects of several anticancer drugs, such as lapatinib, the BET inhibitor JQ1, and PARP inhibitors such as Olaparib.^[^ ^24^, ^48^, ^49^ ^]^ As our results indicate that SCRN2 triggers intrinsic apoptosis and that DNA damage is one of the main causes of the activation of intrinsic apoptosis, we wondered whether SCRN2 affects the DNA damage response and the therapeutic effects of the PARP inhibitor Olaparib. To address this issue, we used phosphorylation of H2AX at Ser 139 (termed γ‐H2AX) as a marker of DNA strand break to detect the DNA damage levels.^[^ ^50^ ^]^ As shown in **Figure** **7**A, the levels of γ‐H2AX were increased in SCRN2‐overexpressing cells and decreased when KMT2C was knockdown. Consistently, γ‐H2AX foci were increased in SCRN2‐overexpressing cells, but this phenomenon was impaired by knockdown of KMT2C in SCRN2‐overexpressing cells (Figure 7B), indicating that SCRN2 stimulated endogenous DNA damage through KMT2C. Next, we treated SCRN2‐overexpressing cells with different doses of Olaparib and found that the suppressive effects on cell survival and viability were more obvious in SCRN2‐overexpressing cells than in control cells (Figure 7C–E). To confirm these results, we generated mouse xenograft tumor models and treated them with saline and Olaparib, respectively. As shown in Figure 7F,G, the suppressive effects of Olaparib on tumor growth were more pronounced in mice bearing SCRN2‐overexpressing tumors than in those bearing tumors expressing the empty vector. Therefore, SCRN2 sensitizes TNBC cells to Olaparib both in vitro and in vivo.

It was reported that KMT2C participates in DNA damage repair by regulating the expression of DNA damage repair‐associated genes.^[^ ^49^ ^]^ To confirm these results, we determined the protein levels and activation status of several DNA damage repair‐associated genes. As shown in Figure 7H and Figure S15 (Supporting Information), both the protein levels and activation status of these genes were suppressed by SCRN2, and the suppressive effects were partially dependent on KMT2C. Taken together, these results suggest that SCRN2 and KMT2C promote DNA damage and sensitize TNBC cells to Olaparib both in vitro and in vivo probably by inhibiting the expression and activation of DNA damage repair genes.

## 3 Discussion

In this study, we report several key findings concerning the previously unknown role of SCRN2 in TNBC progression and the therapeutic responsiveness to PARP inhibition (Figure 7I).

First, we report for the first time that SCRN2 functions as a novel suppressor of TNBC. Very limited information indicates that SCRN2 is associated with neurodegenerative disease and respiratory infectious diseases, and may act as an electrophilic cofactor.^[^ ^5^, ^17^, ^18^ ^]^ However, its functional and mechanistic roles in human cancers have not yet been explored. Analysis of TNBC proteomic datasets from the FUSCC and the CPTAC and immunoblotting validation using clinical TNBC samples showed that SCRN2 was downregulated in TNBC, and its downregulation was correlated with unfavorable prognosis of patients (Figure 1). A series of in vitro and in vivo functional assays with overexpression or knockdown of SCRN2 demonstrated that SCRN2 inhibited TNBC cell proliferation, migration and invasion in vitro, and impeded xenograft tumor growth and lung metastasis in mice (Figures 3 and 5). Additionally, SCRN2 overexpression induced endogenous DNA damage and sensitized TNBC cells to the PARP inhibitor, Olaparib, both in vitro and in vivo. These results collectively suggest that SCRN2 suppresses TNBC progression and that patients with relatively higher expression of SCRN2 may response better to Olaparib or other DNA damage‐based therapies.

Second, we identified the mechanism underlying SCRN2 downregulation in TNBC cells. Analysis of public datasets revealed that genetic mutations, copy number deletions, and promoter DNA methylation were not the major mechanisms underlying SCRN2 downregulation in TNBC. By performing biochemical and molecular assays, we further demonstrated that SCRN2 was degraded by the CMA pathway, and that this process was mediated by HSPA8. These findings partially explain the low expression of SCRN2 in TNBC.

Third, we discovered that SCRN2 exerts its tumor‐suppressive functions by stabilizing the putative tumor suppressor KMT2C. It has been widely documented that KMT2C regulates cancer progression and drug resistance, and genetic mutation leads to loss of its function and expression in human cancers.^[^ ^24^, ^48^, ^51^, ^52^ ^]^ However, the mechanisms governing KMT2C stability are poorly understood. In this study, we demonstrated that SCRN2 interacts with BAG2, which interferes with the interaction of the E3 protein ligase CHIP with KMT2C, thus impeding the ubiquitination and proteasomal degradation of KMT2C (Figure 4). These findings are the first to reveal the mechanisms that regulate KMT2C protein stability and provide a reasonable explanation for the low expression of KMT2C in TNBC.

Fourth, SCRN2 induced endogenous DNA damage and sensitized TNBC cells to PARP inhibition (Figure 7). These results suggest that SCRN2 may function as a potential biomarker for selecting suitable patients with relatively high SCRN2 expression for PARP inhibitor treatment. In addition, direct upregulation of SCRN2 expression using adenovirus or lentiviral vectors, or indirect restoration of SCRN2 expression by inhibiting HSPA8‐mediated CMA degradation using siRNA or small molecular inhibitors targeting HSPA8, may suppress TNBC progression and enhance therapeutic sensitivity to PARP inhibition.

In conclusion, the findings presented here revealed the functions of SCRN2 as a novel tumor suppressor in TNBC and highlighted its potential implication of SCRN2 as a therapeutic target for TNBC. Notably, SCRN2 was downregulated in several types of cancer in the CPTAC dataset (Figure S16, Supporting Information), indicating that our findings are not limited to TNBC. It is reasonable to speculate that SCRN2 may also act as a tumor suppressor in other types of cancers, which should be explored in the near future.

## 4 Experimental Section

### Cell Culture and Reagents

Human embryonic kidney 293T (HEK293T) cells, human mammary epithelial cells (HMECs), and seven representative TNBC cell lines were obtained from the Cell Bank of the Chinese Academy of Sciences (Shanghai, China) and Shanghai Key Laboratory of Breast Cancer (Shanghai, China). HMEC cells were maintained in Dulbecco's Modified Eagle medium (DMEM, #L110KJ, BasalMedia, Shanghai, China) medium supplemented with 5% donor horse serum (#16050‐114, Gibco, Grand Island, NY, USA), 20 ng/mL epidermal growth factor (#10605‐HNAE, Sino Biol, Shanghai, China), 0.5 µg mL^−1^ hydrocortisone (#40109ES08, Yeasen Biotech, Shanghai, China), and 10 µg mL^−1^ insulin (#40107ES76, Yeasen Biotech, Shanghai, China). Other cell lines were maintained in DMEM supplemented with 1% penicillin‐streptomycin (#S110B, BasalMedia, Shanghai, China) and 10% fetal bovine serum (#10270‐106, Gibco, Grand Island, NY, USA). All cell lines were maintained in 5% CO_2_ at 37 °C. All agents used in this study are listed in Table S1 (Supporting Information). All cells were authenticated through short tandem repeat profiling and used for less than 6 months within 15–20 passages. Mycoplasma was tested via PCR using the primers listed in Table S2 (Supporting Information).

### Clinical Samples

Clinical samples were obtained from patients with primary TNBC who underwent surgery at Fudan University Shanghai Cancer Center (FUSCC)(2309‐ZZK‐97). None of the patients received any therapies prior to surgery. Adjacent normal tissues were histologically confirmed to be tumor‐free. The procedures were performed in accordance with the Code of Ethics of the World Medical Association (Declaration of Helsinki) and were approved by the Medical Ethics Committee of FUSCC. Written informed consent was obtained from all patients.

### DNA Constructs, Short Hairpin RNAs (shRNAs), DNA Transfection, and Lentiviral Infection

DNA sequences encoding the indicated genes were amplified by PCR from genomes, and then subcloned into pCDH‐CMV‐MCS‐EF1‐Puro or pLVX‐IRES‐Neo lentiviral vectors. shRNA sequences were obtained from Sigma‐Aldrich (St. Louis, MO, USA), and cloned into the pLKO.1‐TRC vector (#10878, Addgene, Cambridge, MA, USA). All sequences of the molecular cloning primers, shRNAs, and siRNAs used in this study are listed in Tables S2–S5 (Supporting Information).

Neofect DNA transfection reagent (#TF201201, Tengyi Biol, Shanghai, China) was used to transfect the indicated plasmids into HEK293T cells. The supernatants of transfected HEK293T cells were used to infect target cells with 8 µg mL^−1^ polybrene (#H9268, Sigma‐Aldrich, St. Louis, MO, USA). After 48 h of infection, 2 µg mL^−1^ puromycin (#S7417, Selleck Chemicals, Houston, TX, USA) or 0.5 mg mL^−1^ G418 (#A600958‐0005, Sangon Biotech, Shanghai, China) was used to select the infected cells.

### RNA Extraction and Quantitative Real Time‐PCR Assays

TRIzol reagent (#15596018, Invitrogen, Carlsbad, CA, USA) was used to extract total RNA from cultured cells. cDNA was generated from the isolated RNA using a HiScript III RT SuperMix (#R323‐01, Vazyme, Nanjing, Shanghai). Chamq Universal SYBR qPCR Master Mix (#Q711‐03, Vazyme, Nanjing, Shanghai) was used to perform qPCR, and relative gene expression levels among different samples were calculated using the 2^−ΔΔCT^ method. The primers used for qPCR are listed in Table S6 (Supporting Information).

### Antibodies, Immunoblotting, and Immunoprecipitation (IP) Assays

Detailed information of all antibodies used in this study is presented in Table S7 (Supporting Information). For immunoblotting, proteins were extracted from cultured cells using RIPA buffer supplemented with phosphatase and protease inhibitor cocktails (#B15003 and #B14002, Bimake, Shanghai, China). Equal amounts of proteins were subjected to SDS‐PAGE and transferred onto PVDF membranes (#IPVH00010, Meck Millipore, Billerica, MA, USA). Membranes were incubated with appropriate antibodies after blocking with 5% bovine serum albumin (#V900933, Sigma‐Aldrich, St. Louis, MO, USA), and proteins were detected using Chemistar High‐sig ECL kits (#180‐5001E, Tanon, Shanghai, China). For IP assays, cells were lysed using NP40 lysis buffer. Equal amounts of cellular extracts were immunoprecipitated with the indicated antibodies at 4 °C overnight. Immunoprecipitated proteins were subjected to immunoblotting after washing thrice with NP40 lysis buffer.

### Cell Proliferation and Colony Formation Assays

For the cell proliferation assays, cells were plated in 96‐well plates and each experimental group had five replicates. Cell Counting Kit‐8 (CCK‐8) kit (#40203ES92, Yeasen Biotech, Shanghai, China) was used to detect cell proliferation rates according to the manufacturer's protocol. For colony formation assays, cells were plated in 6‐well plates. Each experimental group comprised three replicates. After 14 days of growth, cells were fixed using methanol and stained with 0.2% crystal violet.

### Cell Migration and Invasion Assays

Migration or matrigel‐coated invasion chambers were plated in 24‐well plates containing DMEM supplemented with 10% fetal bovine serum. Cells were re‐suspended in serum‐free DMEM and seeded in the upper chambers. After 24 h of migration or invasion, cells were fixed with methanol and stained with 0.2% crystal violet.

### Xenograft Tumor and Lung Metastatic Experiments

All experimental procedures were approved by the Animal Experiments Committee of FUSCC (FUSCC‐IACUC‐2024020). Mycoplasma‐free cells were injected into the mammary fat pad of 6‐week‐old female BALB/c mice. Tumor volume was measured every 2 days after tumor formation. After about 30 days of growth, mice were executed and tumors were collected. To detect the effects of SCRN2 on the sensitivity to Olaparib in vivo, mycoplasma‐free cells were injected into the mammary fat pad of 6‐week‐old female BALB/c mice. Mice were treated with saline or 50 mg kg^−1^ Olaparib through oral gavage when the tumor volume in one of the groups reached 100 mm^3^. After seven treatments, mice were sacrificed and tumors were collected. For experimental lung metastasis assays, mycoplasma‐free cells were injected into the tail veins of 6‐week‐old female BALB/c mice. After ≈60 days, lungs were collected and subjected to hematoxylin‐eosin (HE) staining.

### Flow Cytometry Analysis of Apoptosis

Apoptosis was detected using an Annexin V‐FITC/PI Apoptosis Detection Kit (#40302ES60, Yeasen Biotech, Shanghai, China) following the manufacturer's instructions. Briefly, cells were collected, washed twice with cold PBS, resuspended in binding buffer and stained by Annexin and V‐FITC for 10–15 mins at room temperature. The prepared samples were analyzed using flow cytometry within 1 h.

### Proteomic Assays

To identify SCRN2‐interacting proteins, HEK293T cells stably expressing pLVX and HA‐SCRN2 were lysed in NP‐40 lysis buffer. Equal amounts of protein were subjected to IP assays. The gel was stained with Coomassie Brilliant Blue and subjected to liquid chromatography‐tandem mass spectrometry (LC‐MS/MS) assays (Applied Protein Technology, Shanghai, China). To identify the downstream targets of SCRN2, LM2‐4175 cells stably expressing empty vector pLVX or HA‐SCRN2 were digested and then collected by centrifugation. The cell pellets were subjected to label‐free quantitative proteomic assays (Applied Protein Technology, Shanghai, China).

### RNA‐Sequencing (RNA‐Seq)

Mycoplasma elimination reagent MycAwayTM (#40607ES08, Yeasen Biotech, Shanghai, China) was used to eliminate mycoplasma from cells. LM2‐4175 cells expressing pLVX, HA‐SCRN2, or HA‐SCRN2 plus shKMT2C #1 were subjected to RNA‐seq (OE Biotech, Shanghai, China) assays.

### Chromatin Immunoprecipitation (ChIP) Assays

ChIP assay was performed using a SimpleChIP Enzymatic Chromatin IP Kit (Magnetic Beads) (#9003S, Cell Signaling Technology, Danvers, MA, USA) following the manufacturer's instructions. Briefly, cells were fixed in 1% formaldehyde at room temperature. Glycine solution (10×) was added to quench unreacted formaldehyde. After sonication, the protein‐DNA complexes were immunoprecipitated using an anti‐H3K4me1 antibody (#A2355, Abclonal, Wuhan, China). Normal IgG was used as a negative control. Protein‐DNA complexes were captured using protein A/G magnetic beads. Immunoprecipitated and input DNA samples were purified to examine enrichment by real‐time PCR. The results are presented as corresponding fold changes. Primers used for ChIP assay are listed in Table S8 (Supporting Information).

### Dual‐Luciferase Reporter Assays

A total of 200 ng of expression vector and 5 ng of Renilla expression vector were transfected into cells using a DNA transfection reagent for 48 h. Cells were subjected to luciferase assays using a dual‐luciferase reporter gene assay kit (#11402ES60, Yeasen Biotech, Shanghai, China). Enhancer activities were normalized to the corresponding values of Renilla luciferase.

### Immunofluorescent Staining

Cells were plated onto coverslips (#12‐545‐80, Thermo Fisher Scientific, Waltham, MA, USA) and fixed with 4% paraformaldehyde (#E672002‐0500, Sangon Biotech, Shanghai, China) for 30 mins. After being permeabilized with 0.1% Triton X‐100, cells were blocked for at least 1 h in 5% bovine serum albumin (#36101ES80, Yeasen Biotech, Shanghai, China) and incubated with primary antibodies at 4 °C overnight. Cells were washed thrice with PBST and incubated with the corresponding fluorescent secondary antibodies for 1 h at room temperature. After incubation, cells were washed thrice with PBST and stained with DAPI (#ab104139, Abcam, Cambridge, UK), and photographed using a Leica SP5 confocal microscope.

### Data Mining

The cBioPortal for Cancer Genomics dataset (https://www.cbioportal.org/) was used to analyze the mutation and copy number deletion frequency of SCRN2. The CpG islands on SCRN2 promoter were analyzed using the MethPrimer program (https://www.urogene.org/index.html). DNA methylation levels of SCRN2 promoter in breast cancer cell lines were measured using Cancer Cell Line Encyclopedia (CCLE) database (https://www.broadinstitute.org/ccle/home). DNA Methylation levels of SCRN2 promoter in normal and TNBC tissues were analyzed using UCSC Xena (https://xena.ucsc.edu/).

### Statistical Analysis

All data are presented as mean ± standard deviation (SD) from three independent experiments. Student's *t*‐test was used to analyze the difference between two groups. Pearson's correlation coefficient was used to evaluate the correlation between the two genes in clinical samples. Univariate and multivariate Cox regression analyses were performed to assess the prognostic value. Results were considered statistically significant at *p* < 0.05 (*p < 0.05; **p < 0.01; ***p < 0.001; n.s, not significant).

## Conflict of Interest

The authors declare no conflict of interest.

## Author Contributions

M‐Y.H and J‐Y.C. are co‐first authors and contributed equally to this work. M.Y.H and J.Y.C. M.Y.H.: data curation, formal analysis, investigation, and writing original draft. J.Y.C.: data curation, formal analysis, and investigation. S.Y.Y. and Q.Z.: writing review and editing. F.L.Z. and Y.L.Z.: writing review and editing, and funding acquisition. Z.M.S. and A.Y.C.: clinical specimens and research resources. D.Q.L.: methodology, supervision, funding acquisition, project administration, writing review and editing.

## Supporting information



Supporting Information

## Data Availability

The data that support the findings of this study are available from the corresponding author upon reasonable request.

## References

[1] G. Bianchini , C. De Angelis , L. Licata , L. Gianni , Nature reviews. Clinical oncology 2022, 19, 91. 10.1038/s41571-021-00565-2 34754128

[2] G. Bianchini , J. M. Balko , I. A. Mayer , M. E. Sanders , L. Gianni , Nat. Rev. Clin. Oncol. 2016, 13, 674. 27184417 10.1038/nrclinonc.2016.66 PMC5461122

[3] Y. Li , H. Zhang , Y. Merkher , L. Chen , N. Liu , S. Leonov , Y. Chen , J. Hematol. Oncol. 2022, 15, 121. 36038913 10.1186/s13045-022-01341-0 PMC9422136

[4] T. Q. Gong , Y. Z. Jiang , C. Shao , W. T. Peng , M. W. Liu , D. Q. Li , B. Y. Zhang , P. Du , Y. Huang , F. F. Li , M. Y. Li , Z. L. Han , X. Jin , D. Ma , Y. Xiao , P. Y. Yang , J. Qin , Z. M. Shao , W. Zhu , Cell Rep. 2022, 38, 110460. 35235781 10.1016/j.celrep.2022.110460

[5] X. Wang , Z. Lin , K. A. Bustin , N. R. McKnight , W. H. Parsons , M. L. Matthews , J. Am. Chem. Soc. 2022, 144, 5377. 35235319 10.1021/jacs.1c12748 PMC10159212

[6] M. L. Matthews , L. He , B. D. Horning , E. J. Olson , B. E. Correia , J. R. Yates 3rd, P. E. Dawson , B. F. Cravatt , Nat. Chem. 2017, 9, 234. 28221344 10.1038/nchem.2645 PMC5325178

[7] G. Way , N. Morrice , C. Smythe , A. J. O'Sullivan , Mol. Biol. Cell 2002, 13, 3344. 12221138 10.1091/mbc.E01-10-0094 PMC124164

[8] X. Yang , C. Tohda , Mol. Neurobiol. 2023, 60, 1250. 36437381 10.1007/s12035-022-03125-6

[9] N. Miyoshi , H. Ishii , K. Mimori , M. Sekimoto , Y. Doki , M. Mori , J. Surg. Oncol. 2010, 101, 156. 20039278 10.1002/jso.21459

[10] G. Pires , S. McElligott , S. Drusinsky , G. Halliday , M. C. Potier , T. Wisniewski , E. Drummond , Acta Neuropathol. Commun. 2019, 7, 195. 31796108 10.1186/s40478-019-0848-6 PMC6892024

[11] S. Lin , T. Jiang , Y. Yu , H. Tang , S. Lu , Z. Peng , J. Fan , Disease markers 2015, 2015, 1. 10.1155/2015/230703 PMC4357136 25814779

[12] L. Xiao , T. Zhang , K. Zheng , Q. Xiao , W. Zhang , D. Zhang , D. Wu , C. He , Y. Zhou , Y. Liu , Sci. Rep. 2023, 13, 14922. 37691034 10.1038/s41598-023-41504-8 PMC10493221

[13] Y. Suehara , N. Tochigi , D. Kubota , K. Kikuta , R. Nakayama , K. Seki , A. Yoshida , H. Ichikawa , T. Hasegawa , K. Kaneko , H. Chuman , Y. Beppu , A. Kawai , T. Kondo , J. Proteomics 2011, 74, 829. 21385630 10.1016/j.jprot.2011.02.033

[14] C. Geisler , N. T. Gaisa , D. Pfister , S. Fuessel , G. Kristiansen , T. Braunschweig , S. Gostek , B. Beine , H. C. Diehl , A. M. Jackson , C. H. Borchers , A. Heidenreich , H. E. Meyer , R. Knüchel , C. Henkel , Biomed. Res. Int. 2015, 2015, 1. 10.1155/2015/454256 PMC4312578 25667921

[15] J. H. Lee , J. Y. Yoo , Y. A. You , W. S. Kwon , S. M. Lee , M. G. Pang , Y. J. Kim , Proteomics 2015, 15, 2669. 25886259 10.1002/pmic.201400359

[16] K. A. Bustin , K. Shishikura , I. Chen , Z. Lin , N. McKnight , Y. Chang , X. Wang , J. J. Li , E. Arellano , L. Pei , P. D. Morton , A. M. Gregus , M. W. Buczynski , M. L. Matthews , Mol. Cell. Neurosci. 2023, 125, 103842. 36924917 10.1016/j.mcn.2023.103842 PMC10247460

[17] B. H. Kim , H. Lee , H. Ham , H. J. Kim , H. Jang , J. P. Kim , Y. H. Park , M. Kim , S. W. Seo , Front. Aging Neurosci. 2023, 15, 1278998. 37901794 10.3389/fnagi.2023.1278998 PMC10602697

[18] X. Zhu , Y. Zou , L. Jia , X. Ye , Y. Zou , J. Tu , J. Li , R. Yu , S. Yang , P. Huang , Front. Genet. 2023, 14, 1164274. 37020999 10.3389/fgene.2023.1164274 PMC10067569

[19] L. Wang , Z. Zhao , P. A. Ozark , D. Fantini , S. A. Marshall , E. J. Rendleman , K. A. Cozzolino , N. Louis , X. He , M. A. Morgan , Y. H. Takahashi , C. K. Collings , E. R. Smith , P. Ntziachristos , J. N. Savas , L. Zou , R. Hashizume , J. J. Meeks , A. Shilatifard , Nat. Med. 2018, 24, 758. 29785026 10.1038/s41591-018-0034-6 PMC6055231

[20] H. M. Herz , M. Mohan , A. S. Garruss , K. Liang , Y. H. Takahashi , K. Mickey , O. Voets , C. P. Verrijzer , A. Shilatifard , Genes Dev. 2012, 26, 2604. 23166019 10.1101/gad.201327.112 PMC3521626

[21] D. Hu , X. Gao , M. A. Morgan , H. M. Herz , E. R. Smith , A. Shilatifard , Mol. Cell. Biol. 2013, 33, 4745. 24081332 10.1128/MCB.01181-13 PMC3838007

[22] M. Kawazu , S. Kojima , T. Ueno , Y. Totoki , H. Nakamura , A. Kunita , W. Qu , J. Yoshimura , M. Soda , T. Yasuda , N. Hama , M. Saito‐Adachi , K. Sato , S. Kohsaka , E. Sai , M. Ikemura , S. Yamamoto , T. Ogawa , M. Fukayama , K. Tada , Y. Seto , S. Morishita , S. Hazama , T. Shibata , Y. Yamashita , H. Mano , PLoS Genet. 2017, 13, e1006853. 28636652 10.1371/journal.pgen.1006853 PMC5500377

[23] L. Li , N. Wu , G. Zhuang , L. Geng , Y. Zeng , X. Wang , S. Wang , X. Ruan , X. Zheng , J. Liu , M. Gao , Front. Pharmacol. 2023, 14, 1224828. 37719859 10.3389/fphar.2023.1224828 PMC10502304

[24] J. Cui , C. Zhang , J. E. Lee , B. A. Bartholdy , D. Yang , Y. Liu , P. Erler , P. M. Galbo Jr. , D. Q. Hodge , D. Huangfu , D. Zheng , K. Ge , W. Guo , Nat. Cell Biol. 2023, 25, 145. 36604594 10.1038/s41556-022-01045-0 PMC10003829

[25] Z. Zhang , J. R. Christin , C. Wang , K. Ge , M. H. Oktay , W. Guo , Cell Rep. 2016, 16, 3146. 27653681 10.1016/j.celrep.2016.08.048 PMC5069998

[26] E. Langille , K. N. Al‐Zahrani , Z. Ma , M. Liang , L. Uuskula‐Reimand , R. Espin , K. Teng , A. Malik , H. Bergholtz , S. E. Ghamrasni , S. Afiuni‐Zadeh , R. Tsai , S. Alvi , A. Elia , Y. Lü , R. H. Oh , K. J. Kozma , D. Trcka , M. Narimatsu , J. C. Liu , T. Nguyen , S. Barutcu , S. K. Loganathan , R. Bremner , G. D. Bader , S. E. Egan , D. W. Cescon , T. Sørlie , J. L. Wrana , H. W. Jackson , et al., Cancer discovery 2022, 12, 2930. 36108220 10.1158/2159-8290.CD-21-0865 PMC9812400

[27] M. Seehawer , Z. Li , J. Nishida , P. Foidart , A. H. Reiter , E. Rojas‐Jimenez , M. A. Goyette , P. Yan , S. Raval , M. M. Gomez , P. Cejas , H. W. Long , M. Papanastasiou , K. Polyak , Nat. Cell Biol. 2024, 26, 1165. 38926506 10.1038/s41556-024-01446-3 PMC11251985

[28] Y. Li , Y. Dou , F. D. a. Veiga Leprevost , Y. Geffen , A. P. Calinawan , F. Aguet , Y. Akiyama , S. Anand , C. Birger , S. Cao , R. Chaudhary , P. Chilappagari , M. Cieslik , A. Colaprico , D. C. Zhou , C. Day , M. J. Domagalski , M. Esai Selvan , D. Fenyö , S. M. Foltz , A. Francis , T. Gonzalez‐Robles , Z. H. Gümüş , D. Heiman , M. Holck , R. Hong , Y. Hu , E. J. Jaehnig , J. Ji , W. Jiang , et al., Cancer Cell 2023, 41, 1397. 37582339 10.1016/j.ccell.2023.06.009 PMC10506762

[29] P. M. Das , R. Singhal , Journal of clinical oncology: official journal of the American Society of Clinical Oncology 2004, 22, 4632. 15542813 10.1200/JCO.2004.07.151

[30] S. Peuget , X. Zhou , G. Selivanova , Nature reviews. Cancer 2024, 24, 192. 38287107 10.1038/s41568-023-00658-3

[31] E. Cerami , J. Gao , U. Dogrusoz , B. E. Gross , S. O. Sumer , B. A. Aksoy , A. Jacobsen , C. J. Byrne , M. L. Heuer , E. Larsson , Y. Antipin , B. Reva , A. P. Goldberg , C. Sander , N. Schultz , Cancer Discov. 2012, 2, 401. 22588877 10.1158/2159-8290.CD-12-0095 PMC3956037

[32] L. C. Li , R. Dahiya , Bioinformatics 2002, 18, 1427. 12424112 10.1093/bioinformatics/18.11.1427

[33] M. Gardiner‐Garden , M. Frommer , J. Mol. Biol. 1987, 196, 261. 3656447 10.1016/0022-2836(87)90689-9

[34] M. J. Goldman , B. Craft , M. Hastie , K. Repečka , F. McDade , A. Kamath , A. Banerjee , Y. Luo , D. Rogers , A. N. Brooks , J. Zhu , D. Haussler , Nat. Biotechnol. 2020, 38, 675. 32444850 10.1038/s41587-020-0546-8 PMC7386072

[35] F. Yang , H. Y. Xie , L. F. Yang , L. Zhang , F. L. Zhang , H. Y. Liu , D. Q. Li , Z. M. Shao , Autophagy 2020, 16, 1061. 32401166 10.1080/15548627.2019.1659609 PMC7469550

[36] L. Wang , D. J. Klionsky , H. M. Shen , Nat. Rev. Mol. Cell Biol. 2023, 24, 186. 36097284 10.1038/s41580-022-00529-z

[37] S. Kaushik , A. M. Cuervo , Nat. Rev. Mol. Cell Biol. 2018, 19, 365. 29626215 10.1038/s41580-018-0001-6 PMC6399518

[38] P. O. Seglen , P. B. Gordon , Proc. Natl. Acad. Sci. USA 1982, 79, 1889. 6952238 10.1073/pnas.79.6.1889 PMC346086

[39] S. Eustermann , A. B. Patel , K. P. Hopfner , Y. He , P. Korber , Nat. Rev. Mol. Cell Biol. 2024, 25, 309. 38081975 10.1038/s41580-023-00683-y PMC12303036

[40] B. Schönbühler , V. Schmitt , H. Huesmann , A. Kern , M. Gamerdinger , C. Behl , Int. J. Mol. Sci. 2016, 18, 69. 28042827 10.3390/ijms18010069 PMC5297704

[41] V. Arndt , C. Daniel , W. Nastainczyk , S. Alberti , J. Höhfeld , Mol. Biol. Cell 2005, 16, 5891. 16207813 10.1091/mbc.E05-07-0660 PMC1289430

[42] X. Wang , Y. Li , M. He , X. Kong , P. Jiang , X. Liu , L. Diao , X. Zhang , H. Li , X. Ling , S. Xia , Z. Liu , Y. Liu , C. P. Cui , Y. Wang , L. Tang , L. Zhang , F. He , D. Li , Nucleic Acids Res. 2022, 50, D719. 34669962 10.1093/nar/gkab962 PMC8728189

[43] K. M. Jozwik , I. Chernukhin , A. A. Serandour , S. Nagarajan , J. S. Carroll , Cell Rep. 2016, 17, 2715. 27926873 10.1016/j.celrep.2016.11.028 PMC5177601

[44] A. Local , H. Huang , C. P. Albuquerque , N. Singh , A. Y. Lee , W. Wang , C. Wang , J. E. Hsia , A. K. Shiau , K. Ge , K. D. Corbett , D. Wang , H. Zhou , B. Ren , Nat. Genet. 2018, 50, 73. 29255264 10.1038/s41588-017-0015-6 PMC6007000

[45] F. Grespi , C. Soratroi , G. Krumschnabel , B. Sohm , C. Ploner , S. Geley , L. Hengst , G. Häcker , A. Villunger , Cell Death Differ. 2010, 17, 1672. 20706276 10.1038/cdd.2010.97 PMC2953534

[46] V. Labi , M. Erlacher , S. Kiessling , A. Villunger , Cell Death Differ. 2006, 13, 1325. 16645634 10.1038/sj.cdd.4401940

[47] J. E. Chipuk , T. Moldoveanu , F. Llambi , M. J. Parsons , D. R. Green , Mol. Cell 2010, 37, 299. 20159550 10.1016/j.molcel.2010.01.025 PMC3222298

[48] N. Simigdala , A. Chalari , A. D. Sklirou , E. Chavdoula , G. Papafotiou , P. Melissa , A. Kafalidou , N. Paschalidis , I. S. Pateras , E. Athanasiadis , D. Konstantopoulos , I. P. Trougakos , A. Klinakis , Cell. Mol. Life Sci. 2023, 80, 100. 36933062 10.1007/s00018-023-04734-7 PMC10024673

[49] T. Rampias , D. Karagiannis , M. Avgeris , A. Polyzos , A. Kokkalis , Z. Kanaki , E. Kousidou , M. Tzetis , E. Kanavakis , K. Stravodimos , K. N. Manola , G. E. Pantelias , A. Scorilas , A. Klinakis , EMBO Rep. 2019, 20, 46821. 10.15252/embr.201846821 PMC6399616 30665945

[50] A. Nagelkerke , P. N. Span , Adv. Exp. Med. Biol. 2016, 899, 1. 27325258 10.1007/978-3-319-26666-4_1

[51] K. M. Stauffer , D. L. Elion , R. S. Cook , T. Stricker , Cancer Med. 2021, 10, 7692. 34581028 10.1002/cam4.4285 PMC8559462

[52] R. J. Fagan , A. K. Dingwall , Cancer Lett. 2019, 458, 56. 31128216 10.1016/j.canlet.2019.05.024 PMC6638576

