# Supplementary material for: Secernin‐2 Stabilizes Histone Methyltransferase KMT2C to Suppress Progression and Confer Therapeutic Sensitivity to PARP Inhibition in Triple‐Negative Breast Cancer
Source: Adv Sci (Weinh). 2025 Jan 21;12(10):2413280. doi: 10.1002/advs.202413280 (PMC11905051; doi:10.1002/advs.202413280)
Supplement: Supplementary file 1 — Supporting Information [file ADVS-12-2413280-s001.docx]

**Supporting Information for**

Huang MY, et al. Secernin-2 stabilizes histone methyltransferase KMT2C to suppress progression and confer therapeutic sensitivity to PARP inhibition in triple-negative breast cancer

**This Supplementary Information includes**

Supplementary Fig. S1 to S16

Supplementary Tables S1 to S8

**Supplementary figures and figure legends**

**
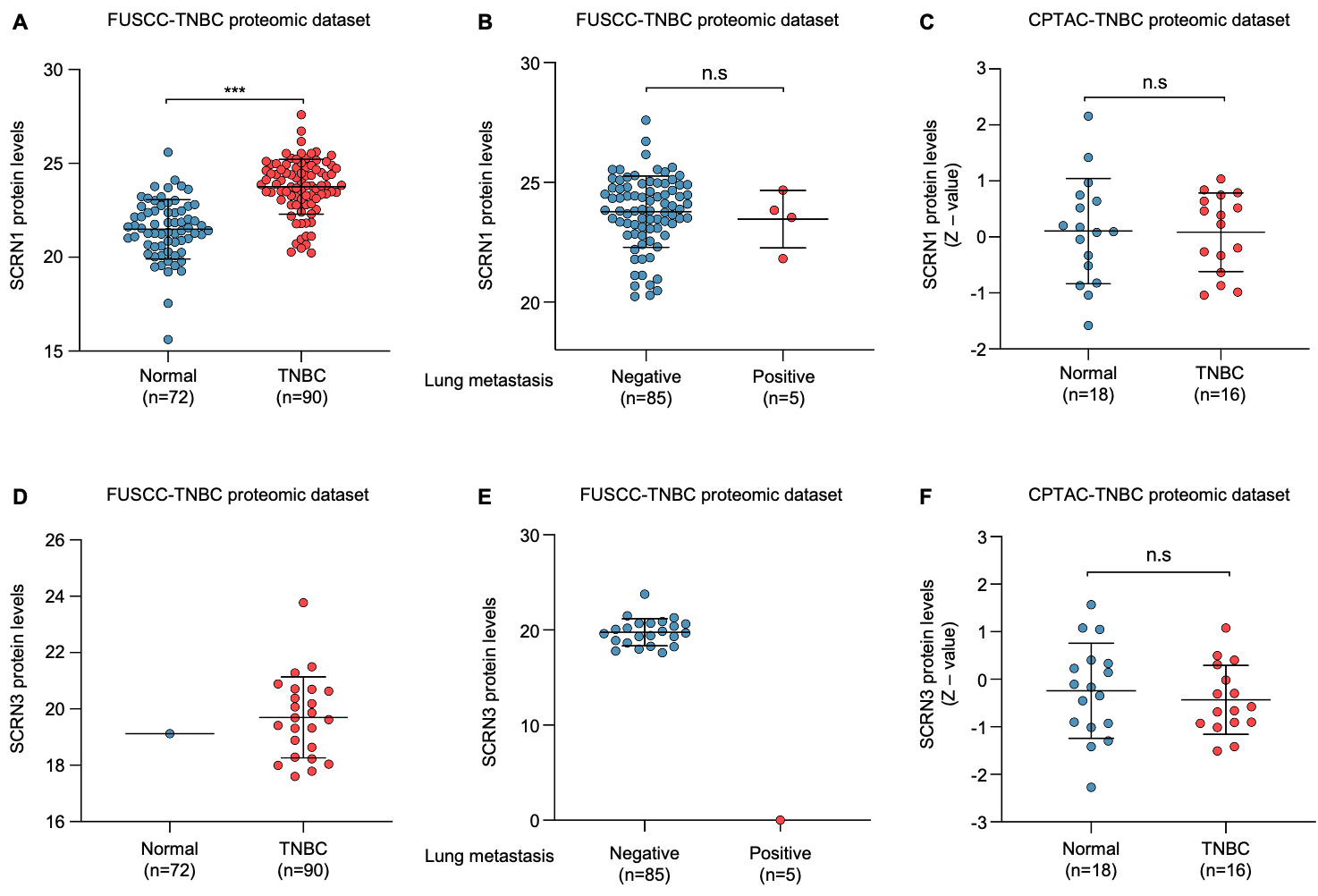
**

**Supplementary Fig. S1. Expression pattens of SCRN1 and SCRN3 in TNBC proteomic dataset from FUSCC and CPTAC**

(A) Expression levels of SCRN1 in normal and TNBC tissues in the FUSCC-TNBC proteomic dataset.

(B) Expression levels of SCRN1 in patients with or without lung metastasis in the FUSCC-TNBC proteomic dataset.

(C) Expression levels of SCRN1 in normal and TNBC tissues in the CPTAC-TNBC proteomic dataset.

(D) Expression levels of SCRN3 in normal and TNBC tissues in the FUSCC-TNBC proteomic dataset.

(E) Expression levels of SCRN3 in patients with or without lung metastasis in the FUSCC-TNBC proteomic dataset.

(F) Expression levels of SCRN3 in normal and TNBC tissues in the CPTAC-TNBC proteomic dataset.

*p* values were calculated using the Student's *t-*test between different group. *, *p* < 0.05, **, *p* <0.01, ***, *p* < 0.001, n.s, not significant.

**
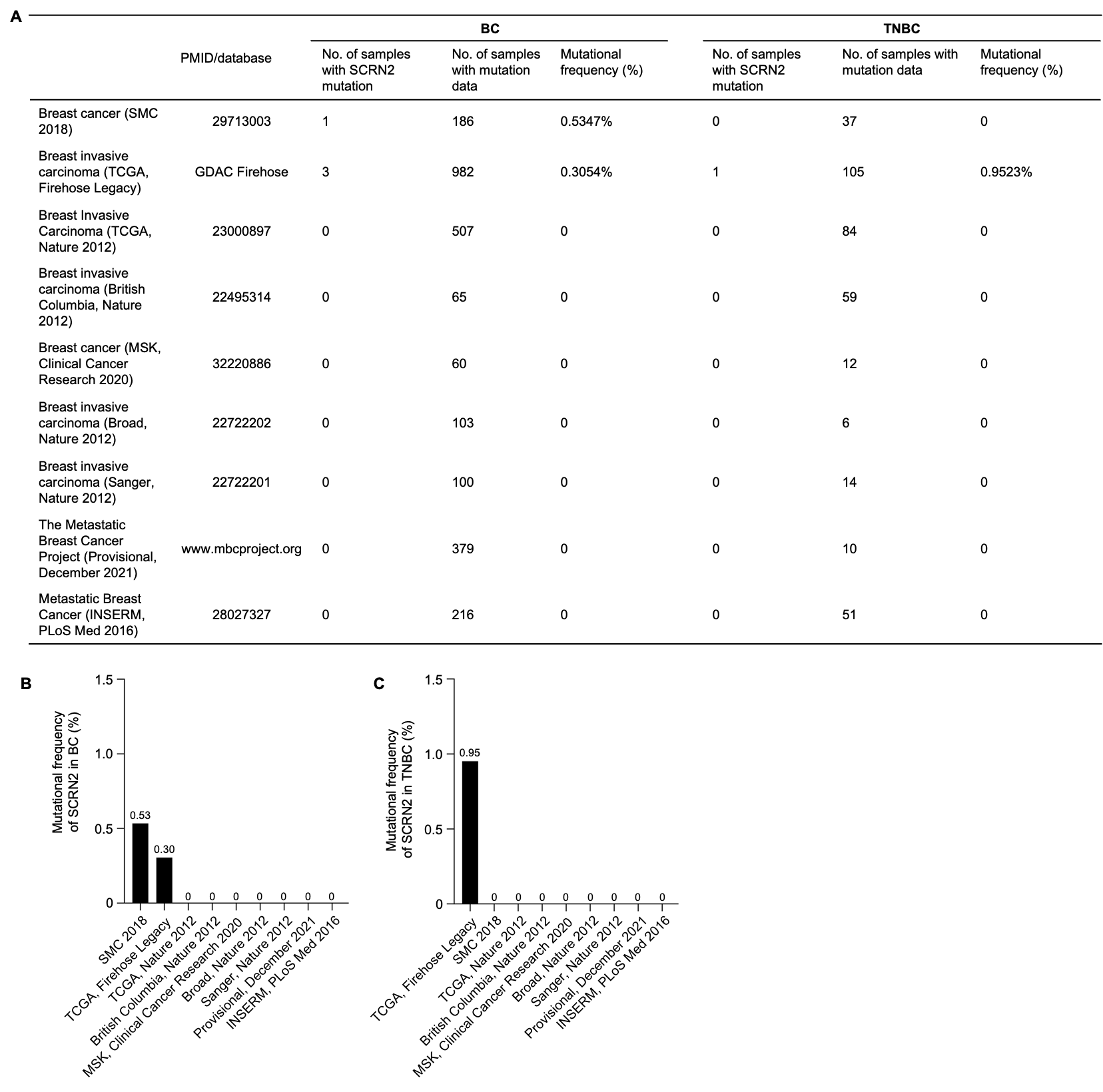
**

**Supplementary Fig. S2. The mutation frequency of SCRN2 in all breast cancer and TNBC**

(A) The datasets used for analysis of SCRN2 mutation frequency in all breast cancer and TNBC from the cBioPortal for Cancer Genomics (https://www.cbioportal.org).

(B and C) Mutation frequency of SCRN2 in all breast cancer (B) and TNBC (C).

**
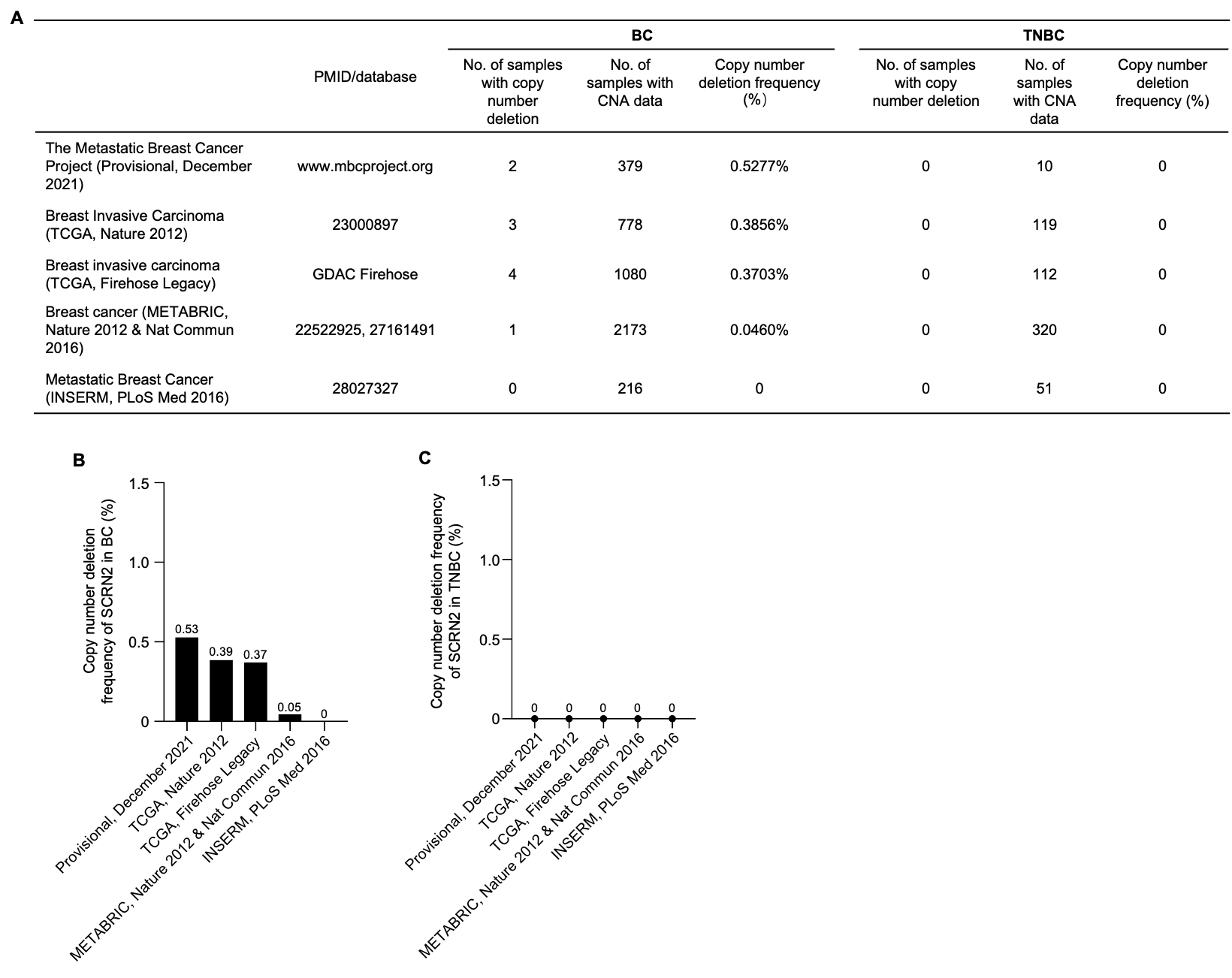
**

**Supplementary Fig. S3. The frequency of copy number deletion of SCRN2** **in all breast cancer and TNBC**

(A) The datasets used for analysis of SCRN2 copy number deep deletion in all breast cancer and TNBC from the cBioPortal for Cancer Genomics (https://www.cbioportal.org).

(B and C) Copy number deep deletion frequency of SCRN2 in all breast cancer (B) and TNBC (C).

**
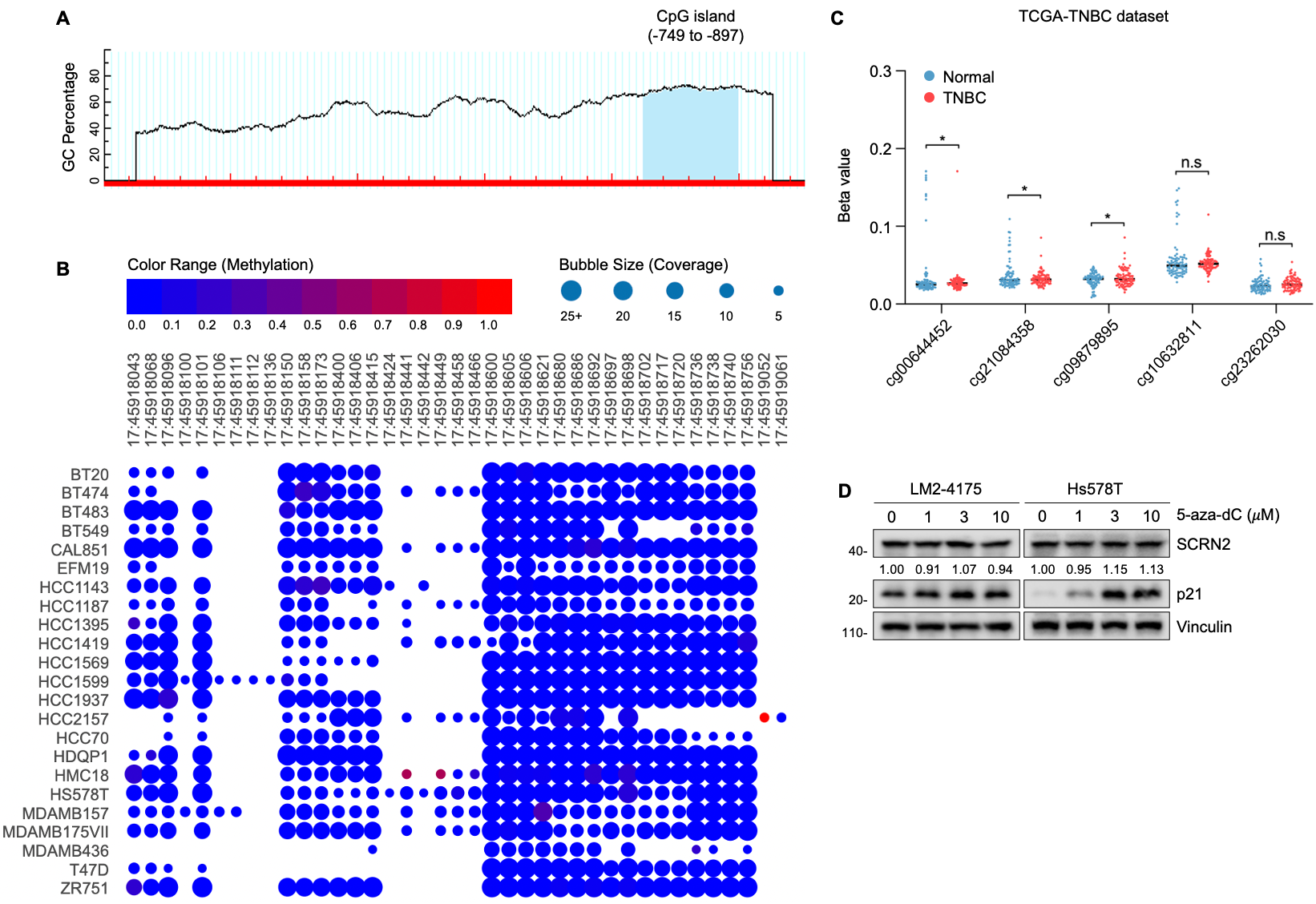
**

**Supplementary Fig. S4. Analysis of the contribution of promoter methylation to SCRN2 downregulation in TNBC**

(A) Analysis of the CpG islands on SCRN2 promoter (from -1000bp to +100bp relative to transcription start site) using MethPrimer program (http://www.urogene.org/index.html) following the established criteria of CpG island size over 100 bp, GC percentage over 50%, and observed/expected CpG ratio over 0.6.

(B) Analysis of DNA methylation levels of SCRN2 promoter in 23 breast cancer cell lines using Cancer Cell Line Encyclopedia (CCLE) dataset (<https://www.broadinstitute.org/ccle/home>).

(C) Analysis of DNA methylation status of SCRN2 promoter in normal and TNBC tissues using UCSC Xena (https://xena.ucsc.edu/).

(D) Immunoblotting analysis of the change of SCRN2 protein levels after treatment with increasing doses of DNA methylation inhibitor 5-aza-dC.

*p* values were calculated using the Student's *t-*test between different group. *, *p* < 0.05, **, *p* <0.01, ***, *p* < 0.001, n.s, not significant.

**
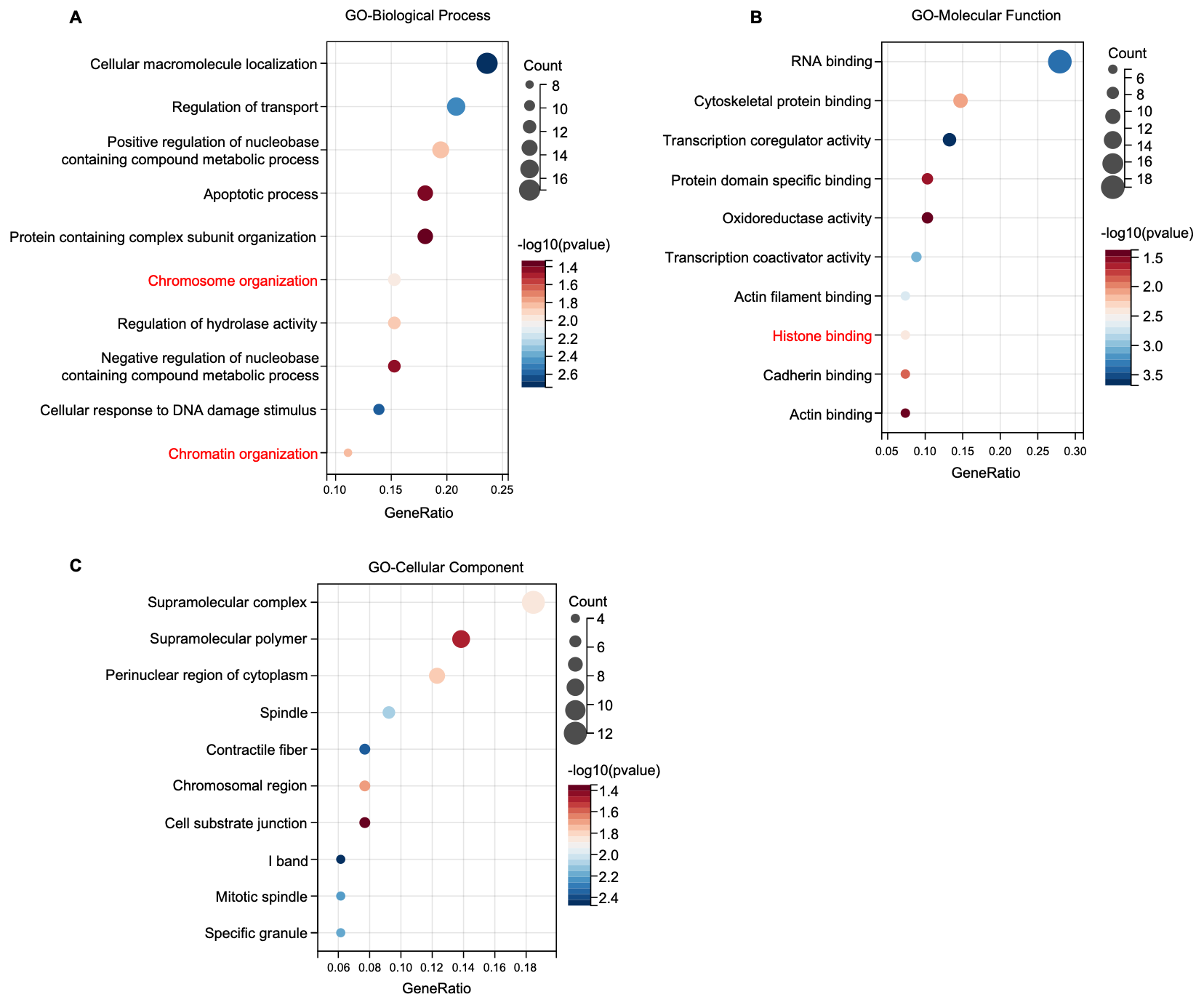
**

**Supplementary Fig. S5. Gene Ontology (GO) analyses of the differentially expressed proteins** **after SCRN2 overexpression**

(A-C) GO analysis of the differentially expressed proteins in the categories of biological process (A), molecular function (B) and cellular component (C).

**
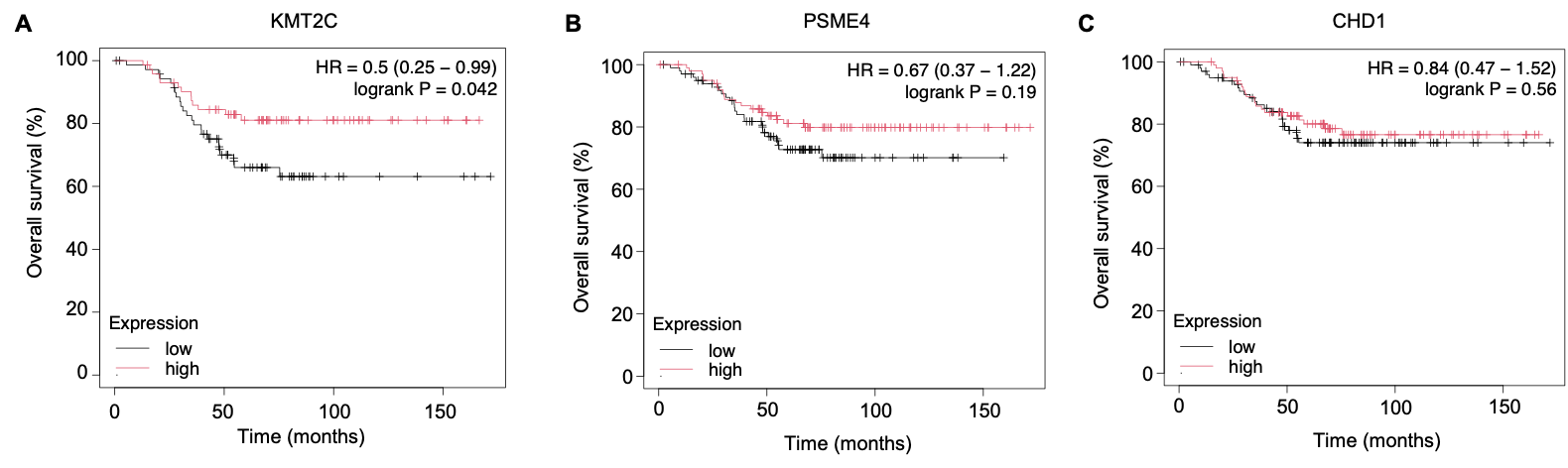
**

**Supplementary Fig. S6. Prognosis value of KMT2C, PSME4 and CHD1 in TNBC patients**

(A-C) Analysis of the overall survival of TNBC patients with high and low expression of KMT2C (A), PSME4 (B), and CHD1 (C) using Kaplan-Meier plotter dataset (https://www.kmplot.com/analysis/).

**
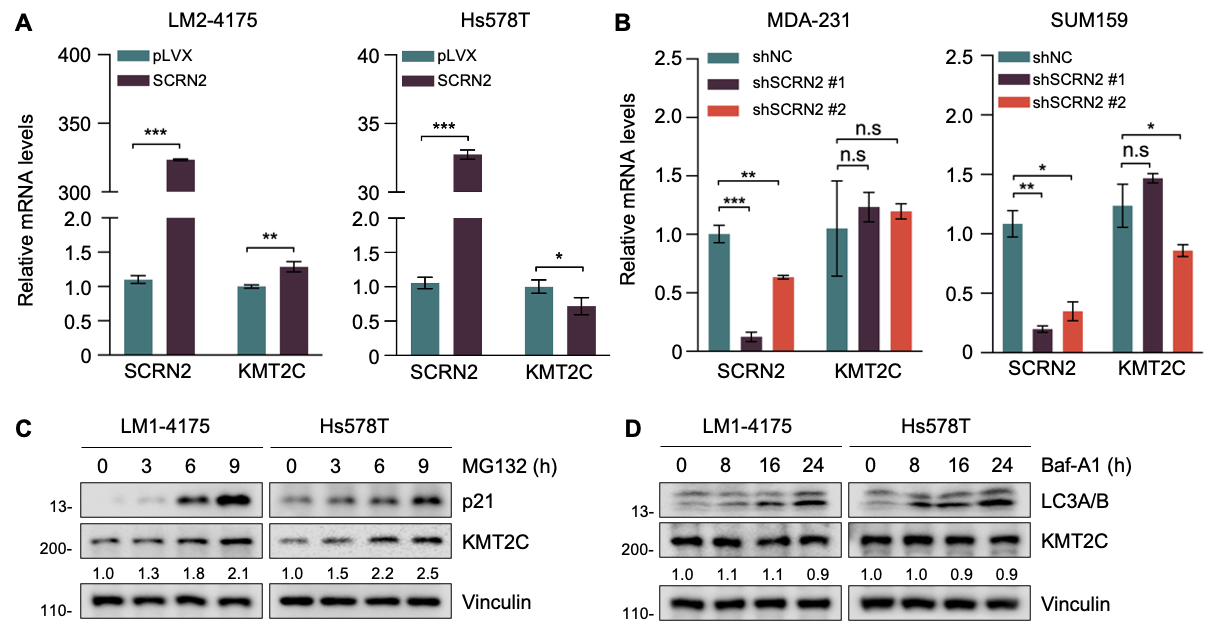
**

**Supplementary Fig. S7. SCRN2 is subjected to degradation through ubiquitin-proteasome pathway**

(A and B) qPCR assays were performed to detect the mRNA levels of KMT2C in SCRN2-overexpressing (A) and -depleted (B) cells.

(C and D) Immunoblotting analysis of the changes of KMT2C protein levels after treatment with MG132 (C) and Baf-A1 (D) for the indicated time.

*p* values were calculated using the Student's *t-*test between different group. *, *p* < 0.05, **, *p* <0.01, ***, *p* < 0.001, n.s, not significant.

**
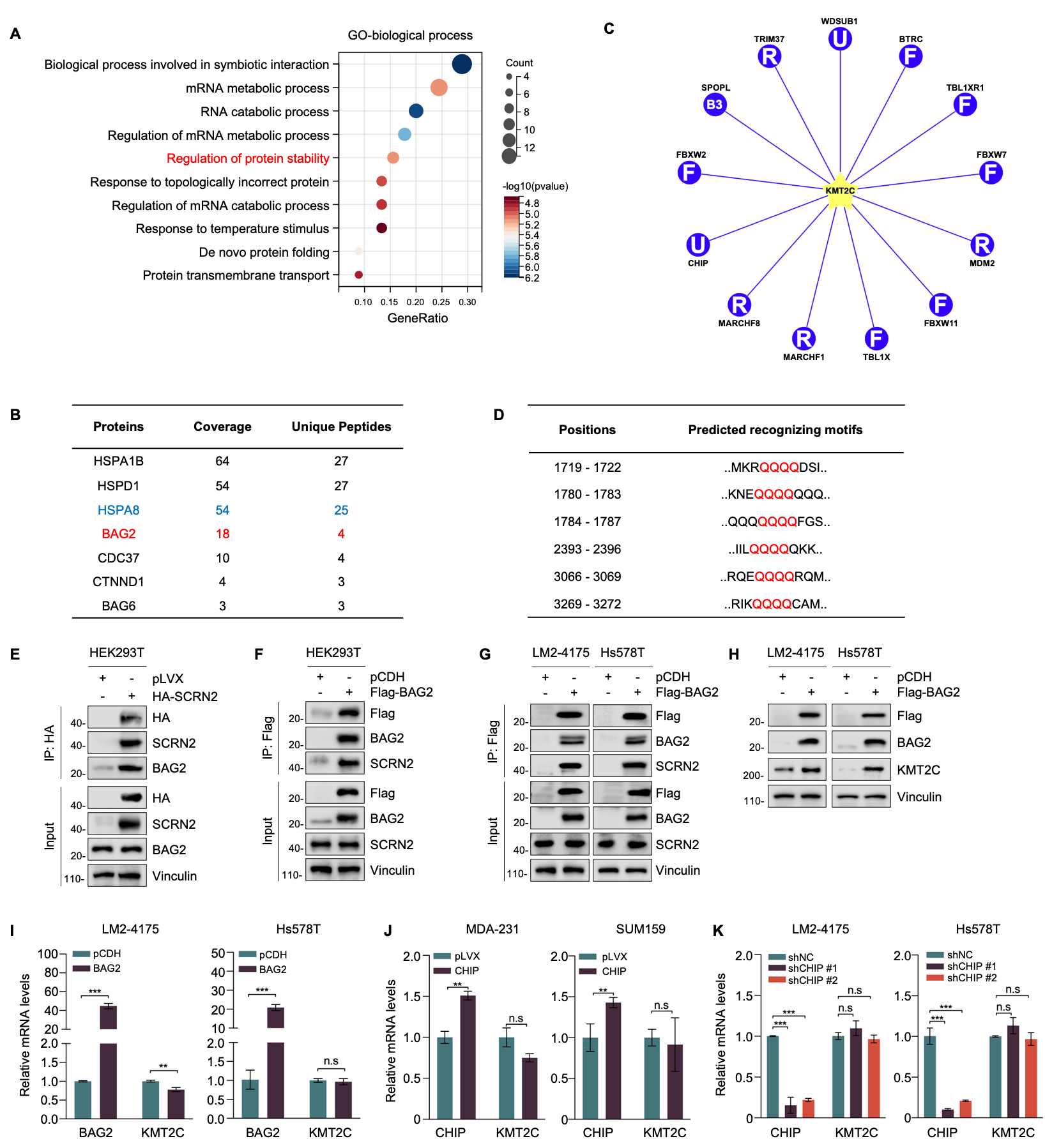
**

**Supplementary Fig. S8. SCRN2 inhibits the ubiquitination of KMT2C through interfering the interaction of E3 ubiquitin-protein ligase CHIP with KMT2C**

(A) HEK293T cells were transfected with pLVX and HA-SCRN2 and then subjected to IP assay using anti-HA affinity gel beads, followed by LC-MS/MS assays to identify the binding partners of SCRN2. The potential binding partners of SCRN2 were determined according to the screening criteria of the identified unique peptides greater than 2, and were subjected to GO-biological process analysis.

(B) The potential binding proteins that are enriched in the pathway of regulation of protein stability through GO-biological process analysis.

(C) The predicted potential E3 ubiquitin ligases of KMT2C using the UbiBrowser dataset (<http://ubibrowser.ncpsb.org.cn>).

(D) Predicted recognizing motifs on KMT2C amino acid sequence by E3 ubiquitin-protein ligase CHIP.

(E) Detection of the interaction of SCRN2 with BAG2 in HEK293T cells by IP assays using anti-HA beads.

(F and G) Detection of the interaction of BAG2 with SCRN2 by IP assays using anti-Flag beads in HEK293T (F) and TNBC (G) cells.

(H) Immunoblotting analysis of the protein levels of KMT2C in BAG2-overexpressing cells.

(I) qPCR assays were performed to detect the mRNA levels of KMT2C in BAG2-overexpressing cells by qPCR assays.

(J and K) qPCR assays were performed to detect the mRNA levels of KMT2C in cells with CHIP overexpression (J) and knockdown (K).

*p* values were calculated using the Student's *t-*test between different group. *, *p* < 0.05, **, *p* <0.01, ***, *p* < 0.001, n.s, not significant.

**
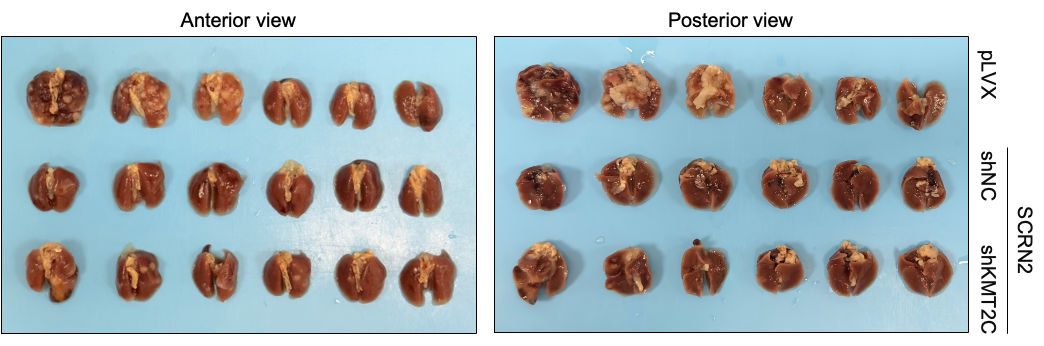
**

**Supplementary Fig. S9. SCRN2 suppresses TNBC metastasis through KMT2C *in vivo***

LM2-4175 cells expressing pLVX, HA-SCRN2 alone or in combination with shKMT2C were injected into the tail vein of 6-week-old BALB/c mice. After about 60 days of metastasis, mice were executed and lungs were removed. The images of collected lungs are shown.

**
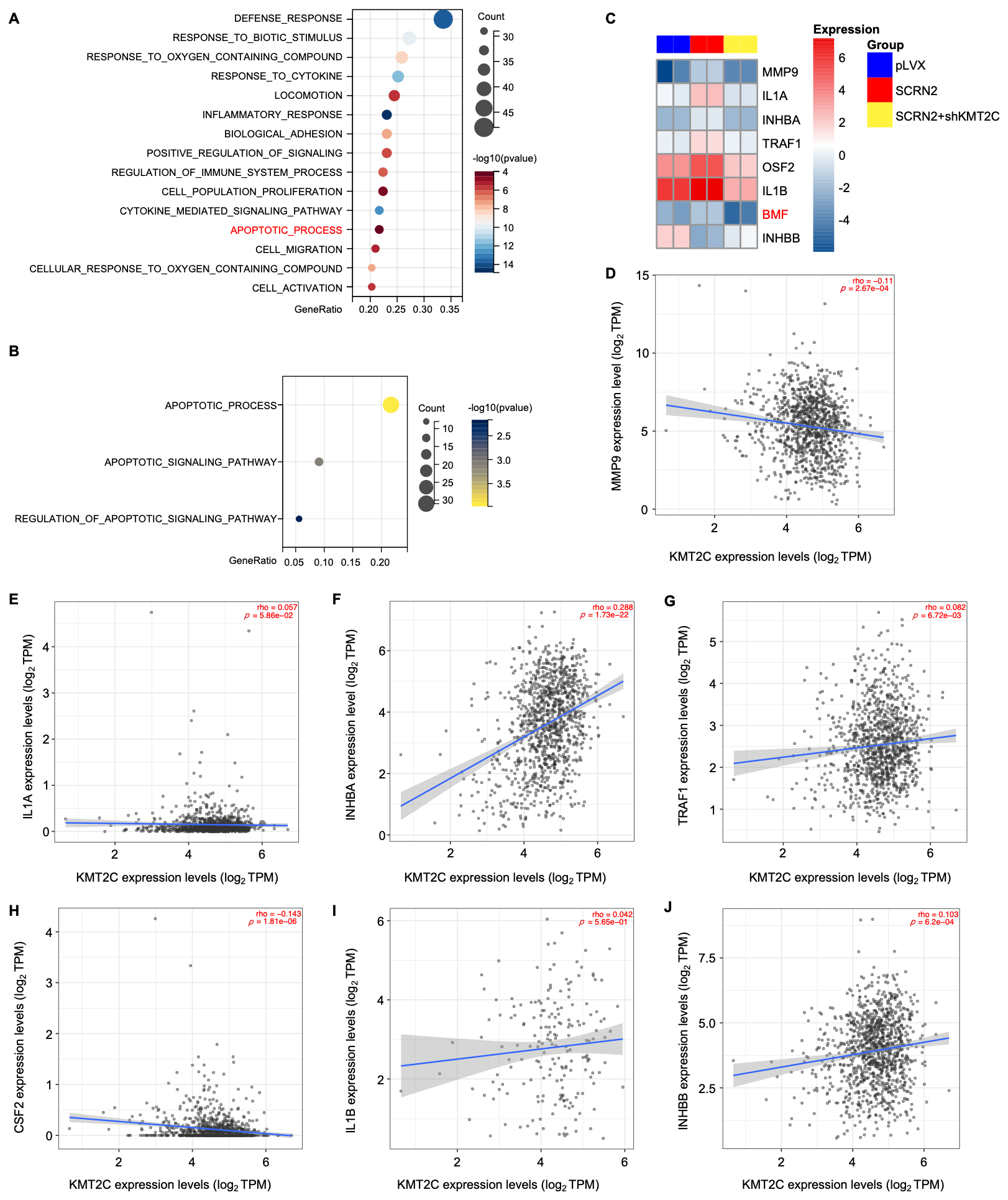
**

**Supplementary Fig. S10. Analysis of differentially expressed genes in** **LM2-4175 cells expressing pLVX, HA-SCRN2 alone or in combination with shKMT2C**

(A) GO-Biological Process analysis of differentially expressed genes in LM2-4175 cells expressing pLVX, HA-SCRN2 alone or in combination with shKMT2C. The apoptotic process pathway was enriched.

(B) Another two apoptosis-associated pathways were enriched in GO-Biological Process analysis.

(C) Expression of the 8 candidate genes obtained by cross-analysis of three apoptosis-associated pathways.

(D-J) Correlation of the expression levels of KMT2C with MMP9 (D), IL1A (E), INHBA (F), TRAF1 (G), CSF2 (H), IL1B (I), INHBB (J) in the TCGA dataset. Data were generated from TIMER 2.0.

**
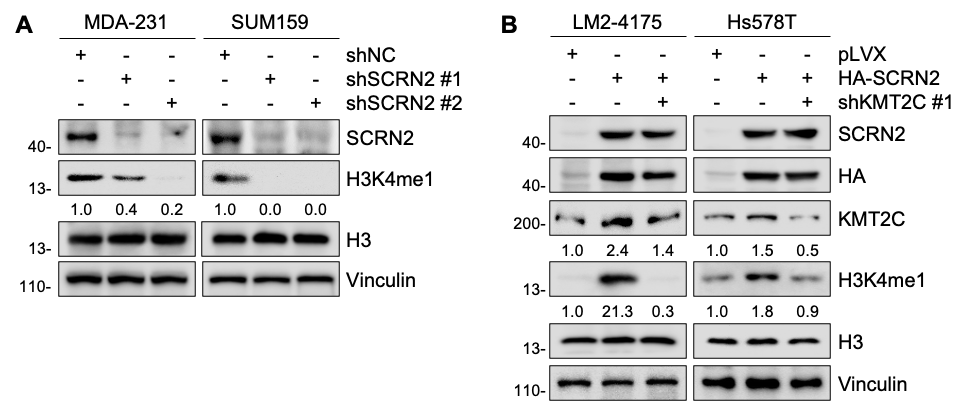
**

**Supplementary Fig. S11. SCRN2 regulates the whole H3K4me1 levels in TNBC cells through KMT2C**

(A and B) Immunoblotting assays were performed to detect the whole H3K4me1 levels in cells overexpressing SCRN2 (A) and in cells expressing pLVX, HA-SCRN2 alone or in combination with shKMT2C (B).

**
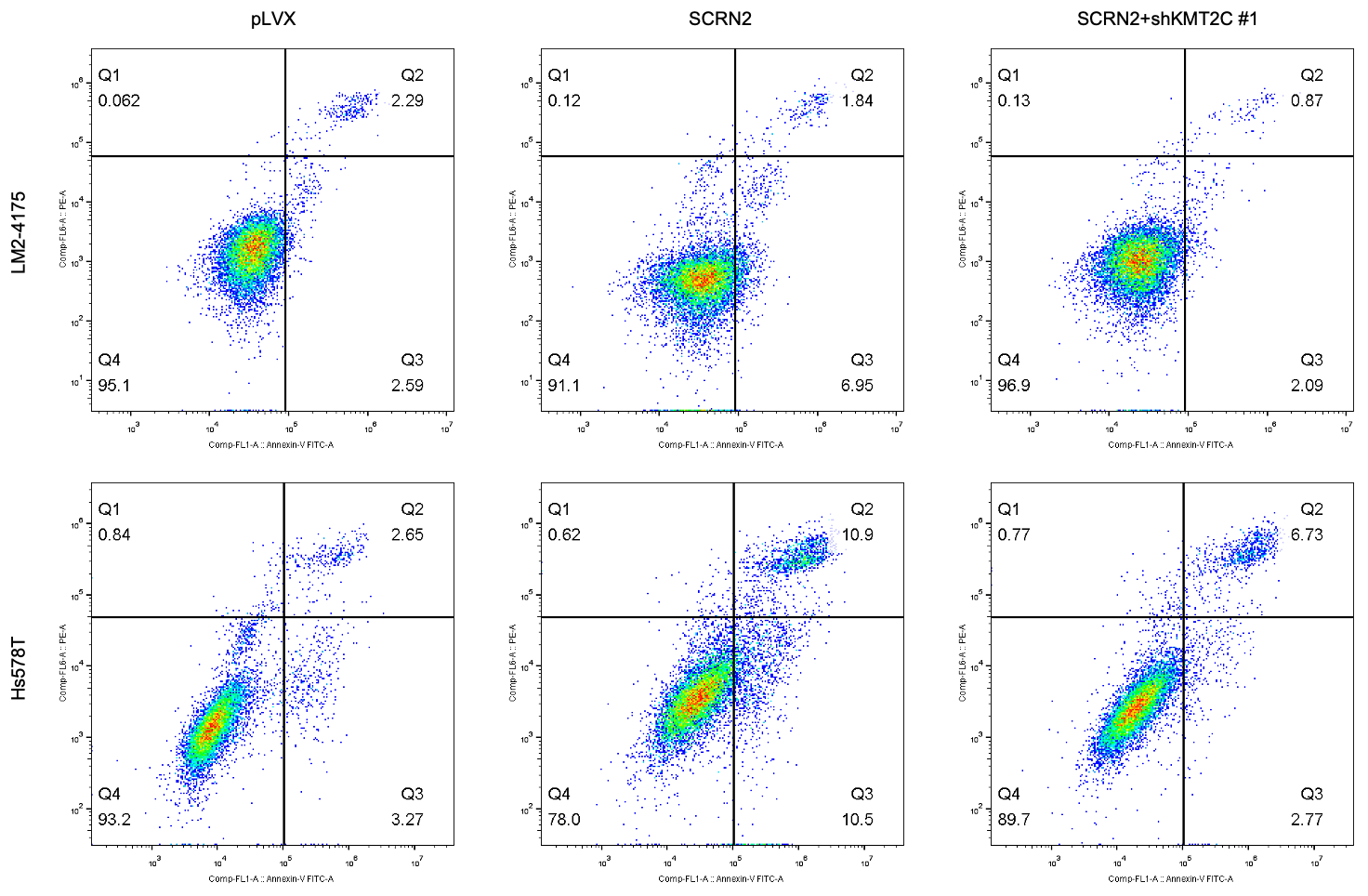
**

**Supplementary Fig. S12. SCRN2 triggers apoptosis through KMT2C**

Apoptosis was detected in cells expressing pLVX, HA-SCRN2 alone or in combination with shKMT2C by flow cytometry analysis.

**
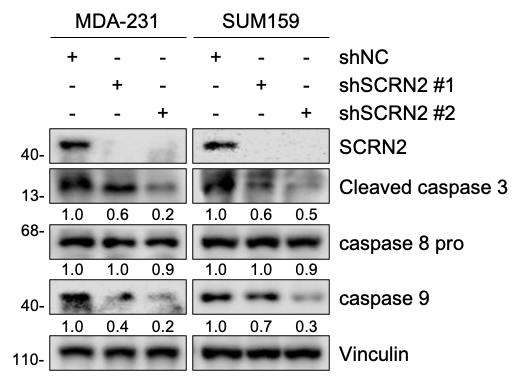
**

**Supplementary Fig. S13. SCRN2 regulates intrinsic apoptosis pathway**

Immunoblotting assays were performed to detect the activation of apoptosis. Cleaved-caspase 3 was used as a marker of the activation of general apoptosis, and caspase 8 pro and caspase 9 were used as a marker of the activation of extrinsic and intrinsic apoptosis pathway, respectively.

**
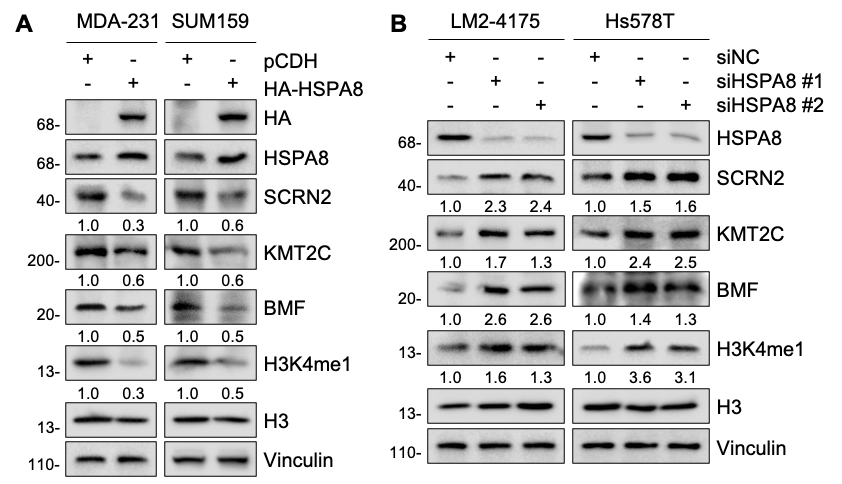
**

**Supplementary Fig. S14. The HSPA8/SCRN2/KMT2C/BMF axis in TNBC**

(A and B) Immunoblotting analysis of the expression levels of HSPA8, SCRN2, KMT2C, BMF, and H3K4me1 in HSPA8-overexpressing (A) and -depleted (B) cells.

**
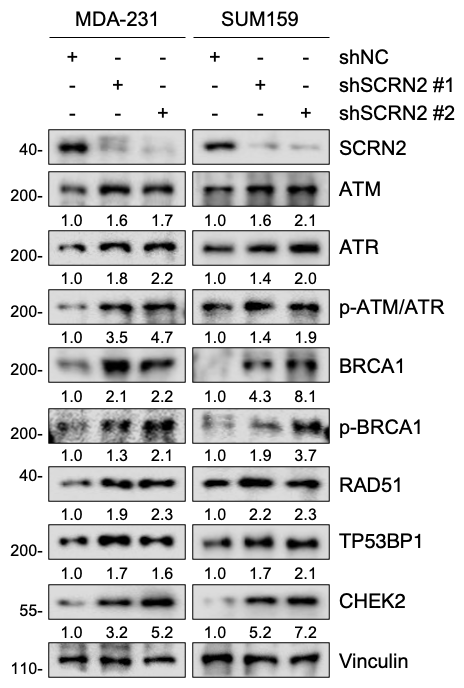
**

**Supplementary Fig. S15. SCRN2 inhibits the expression and activation of DNA damage repair-associated genes**

Immunoblotting analysis of the expression and activation of DNA damage repair-associated genes in SCRN2-knocked down cells.

**
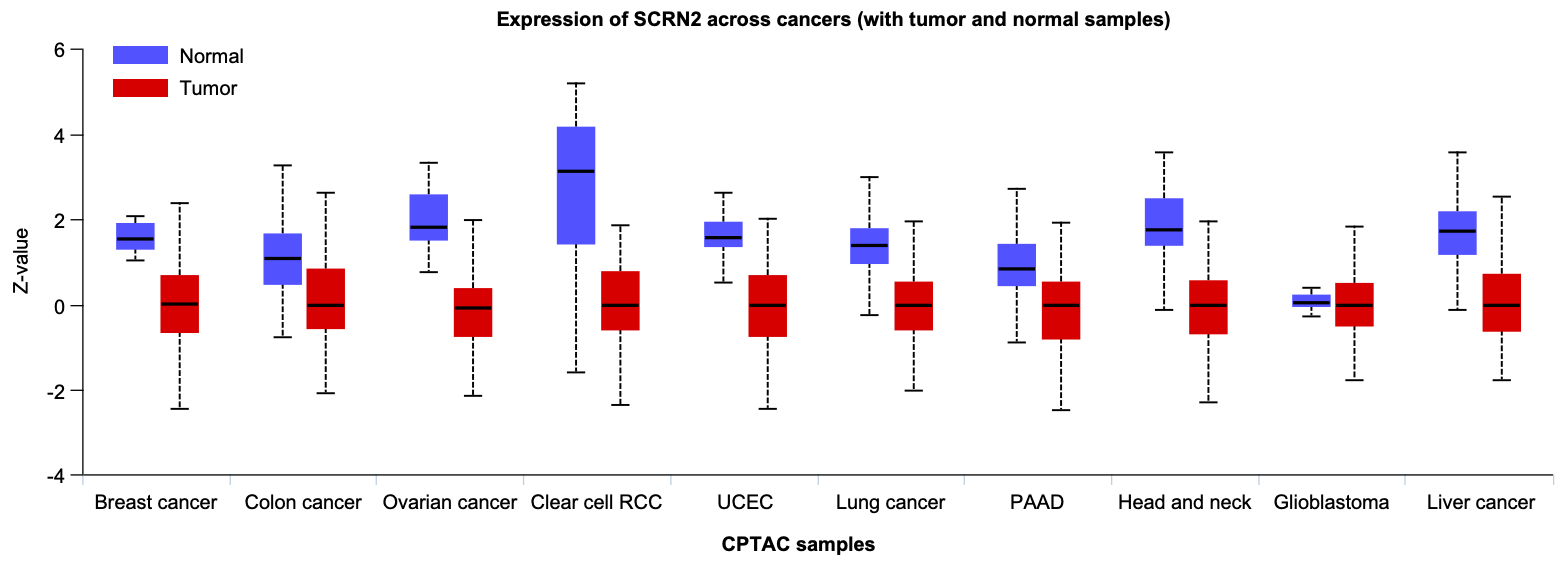
**

**Supplementary Fig. S16. SCRN2 is generally downregulated across different types of cancers**

The protein expression levels of SCRN2 in normal and different types of tumor tissues in the CPTAC dataset.

**Supplementary Tables**

**Supplementary Table S1. Chemical reagents used in this study**

| **Chemical reagents** | **Vendors** | **Cat#** | **Working concentration** |
| --- | --- | --- | --- |
| Cycloheximide (CHX) | CST | 2112S | 100 µg/mL |
| MG132 | Selleck | S2619 | 10 μM |
| Baf-A1 | Selleck | S1413 | 200 nM |
| 3-MA | Selleck | S2767 | 1 mM |
| Rapamycin | Selleck | S1039 | 1 μM |
| Protease inhibitor cocktail | Bimake | B14002 | 1× |
| Phosphatase inhibitor cocktail | Bimake | B15001 | 1× |
| 5-aza-dC | Selleck | S1200 | 1, 3, 10 μM |

**Supplementary Table S2. Primers used for molecular cloning of expression vectors**

| **Plasmids** | **Primers** | **Sequences** |
| --- | --- | --- |
| Flag-SCRN2 | Forward | ACCTCCATAGAAGATTCTAGAGCCACCATGGCGTCGTCGAGCCCTGAC |
|  | Reverse | ATCCGATTTAAATTCGAATTCTTACTTGTCATCGTCGTCCTTGTAATCCGCATAAGCCTGGCTCTCCCT |
| HA-SCRN2 | Forward | GGATCTATTTCCGGTGAATTCGCCACCATGGCGTCGTCGAGCCCTGAC |
|  | Reverse | GGGATCCGCGGCCGCTCTAGATTAAGCGTAGTCTGGGACGTCGTATGGGTACGCATAAGCCTGGCTCTCCCT |
| HA-HSPA8 | Forward | ACCTCCATAGAAGATCTAGAGCCGCCACCATGTCCAAGGGACCTGCAGTTGG |
|  | Reverse | GATCCATTTAAATTCGAATTCTTAAGCGTAATCTGGAACATCGTATGGGTAATCAACCTCTTCAATGGTGGG |
| MYC-CHIP | Forward | GGATCTATTTCCGGTGAATTCGCCACCATGAAGGGCAAGGAGGAGAAG |
|  | Reverse | GGGATCCGCGGCCGCTCTAGATTAGAGCAGAAACTCATCTCTGAAGAGGATCTGGTAGTCCTCCACCCAGCC |
| HA-CHIP | Forward | GGATCTATTTCCGGTGAATTCGCCACCATGAAGGGCAAGGAGGAGAAG |
|  | Reverse | GGGATCCGCGGCCGCTCTAGATTAAGCGTAGTCTGGGACGTCGTATGGGTAGTAGTCCTCCACCCAGCC |
| pGL3-promoter-E1 | Forward | CGAGCTCTTACGCGTGCTAGCAGCAGTGCGATTCCCAAATGG |
|  | Reverse | ACTTAGATCGCAGATCTCGAGGATTCCGACCACCACGCG |
| pGL3-promoter-E2 | Forward | CGAGCTCTTACGCGTGCTAGCCCCAGAGGCAGAGTGCCC |
|  | Reverse | ACTTAGATCGCAGATCTCGAGTATTGAAGGAGACATTGATCT |
| Flag-BAG2 | Forward | ACCTCCATAGAAGATTCTAGAGCCACCATGGCTCAGGCGAAGATCAAC |
|  | Reverse | ATCCGATTTAAATTCGAATTCTTACTTGTCATCGTCGTCCTTGTAATCATTGAATCTGCTTTCAGCATTTTG |
| Mycoplasma | Forward | GGGAGCAAACAGGATTAGATACCCT |
|  | Reverse | TGCACCATCTGTCACTCTGTTAACCTC |

**Supplementary Table S3. Information for expression vectors used in this study**

| **Plasmids** | **Sources** | **Vectors** |
| --- | --- | --- |
| shSCRN2 | Subcloned | pLKO.1-TRC |
| shKMT2C | Subcloned | pLKO.1-TRC |
| HA-SCRN2 | Subcloned | pLVX-IRES-Neo |
| MYC-SCRN2 | Subcloned | pLVX-IRES-Neo |
| HA-CHIP | Subcloned | pLVX-IRES-Neo |
| MYC-CHIP | Subcloned | pLVX-IRES-Neo |
| Flag-SCRN2 | Subcloned | pCDH-CMV-MCS-EF1-Puro |
| Flag-BAG2 | Subcloned | pCDH-CMV-MCS-EF1-Puro |
| HA-HSPA8 | Subcloned | pCDH-CMV-MCS-EF1-Puro |
| pGL3-promoter-E1 | Subcloned | pGL3-promoter |
| pGL3-promoter-E2 | Subcloned | pGL3-promoter |

**Supplementary Table S4. Targeting sequences for shRNAs**

| **shRNAs** | **Primers** | **Sequences** |
| --- | --- | --- |
| shSCRN2 #1 | Forward | CCGGCAGGGTTACTGGAGCACTATGCTCGAGCATAGTGCTCCAGTAACCCTGTTTTTG |
|  | Reverse | AATTCAAAAACAGGGTTACTGGAGCACTATGCTCGAGCATAGTGCTCCAGTAACCCTG |
| shSCRN2 #2 | Forward | CCGGGCTGTGATCTTTGCCAAGAACCTCGAGGTTCTTGGCAAAGATCACAGCTTTTTG |
|  | Reverse | AATTCAAAAAGCTGTGATCTTTGCCAAGAACCTCGAGGTTCTTGGCAAAGATCACAGC |
| shKMT2C #1 | Forward | CCGGCCCTGTTAGAATGCCCAGTTTCTCGAGAAACTGGGCATTCTAACAGGGTTTTTG |
|  | Reverse | AATTCAAAAACCCTGTTAGAATGCCCAGTTTCTCGAGAAACTGGGCATTCTAACAGGG |
| shKMT2C #2 | Forward | CCGGAGCAAGATAAGTTTAGATAATCTCGAGATTATCTAAACTTATCTTGCTTTTTTG |
|  | Reverse | AATTCAAAAAAGCAAGATAAGTTTAGATAATCTCGAGATTATCTAAACTTATCTTGCT |

**Supplementary Table S5. Sequences of siRNAs used in this study**

| **shRNAs** | **Primers** | **Sequences** |
| --- | --- | --- |
| siNC | Forward | TATGCCGCTGTGCTCTAT |
|  | Reverse | TTCTTCACCTCCTGCTCC |
| siLAMP2A #1 | Forward | CGCUAUGAAACUACAAAUATT |
|  | Reverse | UAUUUGUAGUUUCAUAGCGTT |
| siLAMP2A #2 | Forward | GCUCUACUUAGACUCAAUATT |
|  | Reverse | UAUUGAGUCUAAGUAGAGCTT |
| siHSPA8 #1 | Forward | GCAAAGAAUCAAGUUGCAATT |
|  | Reverse | UUGCAACUUGAUUCUUUGCTT |
| siHSPA8 #2 | Forward | GCUGUUGUCCAGUCUGAUATT |
|  | Reverse | UAUCAGACUGGACAACAGCTT |

**Supplementary Table S6. Primers for qPCR analysis**

| **Genes** | **Primers** | **Sequences** |
| --- | --- | --- |
| SCRN2 | Forward | CGAAGACGCACGCTGTGAT |
|  | Reverse | GCAGACACCATGCTCGTTG |
| HSPA8 | Forward | ACCTACTCTTGTGTGGGTGTT |
|  | Reverse | GACATAGCTTGGAGTGGTTCG |
| KMT2C | Forward | CTCGCAAAGATGGCGCTTC |
|  | Reverse | TCTGTTTCCACAATCGTTTCTGT |
| GAPDH | Forward | TCGGAGTCAACGGATTTGGT |
|  | Reverse | TTCCCGTTCTCAGCCTTGAC |
| BMF | Forward | GAGCCATCTCAGTGTGTGGAG |
|  | Reverse | GCCAGCATTGCCATAAAAGAGTC |
| CHIP | Forward | AGCAGGGCAATCGTCTGTTC |
|  | Reverse | CAAGGCCCGGTTGGTGTAATA |

**Supplementary Table S7. Antibodies used in this study**

| **Antibodies** | **Vendors** | **Cat#** | **Hosts** | **Working concentration** |
| --- | --- | --- | --- | --- |
| SCRN2 | Abclonal | A1205 | Rabbit | 1:500 |
| HSPA8 | Abcam | ab51052 | Rabbit | 1:1000 |
| LAMP2A | Abcam | ab125068 | Rabbit | 1:1000 |
| p21 | ZEN-BIOSCIENCE | R382492 | Rabbit | 1:1000 |
| SQSTM1 | Abclonal | A19700 | Rabbit | 1:2000 |
| LC3A/B | CST | 12741 | Rabbit | 1:1000 |
| KMT2C | Proteintech | 28437-1-AP | Rabbit | 1:1000 |
| BAG2 | Abclonal | A19945 | Rabbit | 1:1000 |
| CHIP | ZEN-BIOSCIENCE | R25823 | Rabbit | 1:2000 |
| BMF | Abclonal | A5796 | Rabbit | 1:1000 |
| BMF | Proteintech | 18298-1-AP | Rabbit | 1:500 |
| H3 | Abclonal | A2348 | Rabbit | 1:2000 |
| H3K4me1 | Abclonal | A2355 | Rabbit | 1:2000 |
| HA | Servicebio | GB151252-100 | Mouse | 1:1000 |
| HA | CST | 3724S | Rabbit | 1:3000 |
| Flag | Sigma | F1804 | Mouse | 1:3000/1:200 (IF) |
| MYC-tag | CST | 2276S | Mouse | 1:5000 |
| Vinculin | Sigma | V9131 | Mouse | 1:5000 |

**Supplementary Table S8. Primers for ChIP-qPCR analysis**

| **Names** | **Primers** | **Sequences** |
| --- | --- | --- |
| BMF enhancer #1 | Forward | AGAAAGGAGGGGTGGGGTAG |
|  | Reverse | CGGCCTTGCAGGATTAAAGG |
| BMF enhancer #2 | Forward | GTCAGACCACCTTCCACCTG |
|  | Reverse | ACAGCAGCCACTATTCACCC |
